# Eye Tracking–Based Evaluation of Auditory Spatial Attention in a Virtual Auditory Environment: Protocol for Development of a New Approach and Preliminary Validation

**DOI:** 10.2196/88231

**Published:** 2026-09-29

**Authors:** Clémence Lelaumier, Franck Doidy, Baptiste Fruleux, Antoine Petroff, Valentin Bauer, Hervé Platel

**Affiliations:** 1 Université de Caen Normandie, Inserm, EPHE-PSL, PSL University Paris, CHU de Caen, GIP Cyceron, U1077, NIMH, 2, Rue des Rochambelles, Caen, F-14032, France, 33(0)231568379; 2 Wivy, Lille, France; 3 Cosmos acoustique, Paris, France; 4 Sciences et Technologies de la Musique et du Son (STMS Lab), IRCAM, CNRS, Sorbonne Université, Ministère de la Culture, Paris, France

**Keywords:** auditory spatial attention, oculometry, pupillometry, auditory virtual reality, head tracking, sound localization task.

## Abstract

**Background:**

Auditory spatial attention (ASA) can be impaired in clinical populations and affects everyday activities, yet it remains understudied and lacks practical assessment tools for clinical use. However, recent technological advances have enabled the development of auditory virtual environments capable of engaging attentional networks while remaining compatible with clinical implementation and supporting the collection of objective physiological measures.

**Objective:**

This study aimed to address the lack of clinically applicable ASA assessments by developing a new assessment (phase 1) and supporting its future implementation in clinical practice through preliminary validation and user experience evaluation (phase 2).

**Methods:**

In phase 1, the assessment was developed through a multidisciplinary research committee involving clinicians and researchers, and was refined through pilot testing with 17 healthy participants. It uses an immersive auditory virtual environment and combines eye tracking, head tracking, and pupillometry. Phase 2 will consist of two successive studies: (1) 15 expert clinicians (neuropsychologists or speech therapists) will evaluate face and content validity, and user experience, and (2) 80 healthy adults will provide additional sources of validity evidence and user experience data. Participants will complete 5 steps of an ASA task, alternating habituation and measurement blocks, using either gaze orientation or head orientation as the response modality. Vocal targets will be presented with or without background sound in a dynamic binaural environment.

**Results:**

Study 1 will provide preliminary validity evidence based on face and content, together with user experience outcomes, including usability, satisfaction, and intention to use. Recruitment of participants began in November 2025 and was completed in January 2026. Data analysis is in progress, and the corresponding manuscript is expected to be submitted for publication during the winter of 2026‐2027. Study 2 will provide preliminary validity evidence based on precision, face and content, response processes, internal structure, and fairness, together with user experience outcomes, including usability, satisfaction, and sense of presence. Recruitment of participants began in March 2026 and is expected to be completed in October 2026. As of July 2026, 48 participants had been enrolled. Completion of data analysis and submission of the corresponding manuscript are expected in winter 2027‐2028.

**Conclusions:**

This validation study represents the first step toward developing a clinically applicable tool for assessing ASA. By combining immersive auditory technology with clinically feasible hardware, the proposed approach aims to provide a rapid and ecologically valid evaluation of ASA. Future studies will focus on formal psychometric validation, patient testing, and the development of normative data.

## Introduction

### Background

Auditory spatial attention (ASA) is fundamental because it forms the basis for a number of everyday activities, such as crossing the street while adjusting to the sounds of cars or communicating with different interlocutors. ASA can be defined as “the ability to focus auditory perception on the specific location of a sound source in the environment” [1]. It involves at least 2 levels: orienting attention toward a single sound source and selectively focusing on a target sound arising from a complex spatialized auditory environment [2]. However, ASA is impaired in several pathologies. In schizophrenia, for example, ASA is hindered by excessive attraction to auditory spatial information coming from the left side of space [3]. Similarly, stroke can lead to auditory unilateral spatial neglect, characterized by an attentional bias toward the ipsilesional space [4]. Thus, the potential prevalence of ASA impairments requires dedicated assessments. Moreover, relying solely on measures of visual spatial attention may not be sufficient, as auditory and visual spatial attention rely partially on different brain mechanisms [2] and may therefore be selectively impaired.

Nevertheless, assessing ASA during neuropsychological evaluation in patients with neurological and psychiatric disorders can be challenging. Indeed, ASA assessment requires ecologically valid auditory environments, both in terms of sound content and spatial rendering, in order to effectively engage the cognitive and neural processes underlying ASA. In particular, everyday familiar sounds must be used, as the nature of the sounds can influence auditory attention orienting [5]. Furthermore, several studies suggest that the engagement of ASA depends on the ability to construct a coherent representation of the auditory environment, which requires listeners to perceive sound sources as originating from different regions of auditory space [6]. To provide such spatially distributed sound sources, many experimental paradigms rely on complex spatial-audio setups, including loudspeaker arrays requiring precise spatial positioning [1,7] and sound-attenuated booths [1,8], which limit their implementation in clinical settings.

Experimental paradigms to assess ASA developed in research settings generally require participants to process the spatial attributes of sounds, either by localizing sounds or by selectively attending to sounds originating from a particular region of auditory space. To report their responses, these studies frequently rely on upper limb motor actions [7,9-11]. Beyond potential difficulties in patients with motor impairments, fixed response device positions and constrained upper limb responses directed toward predetermined spatial locations may influence task performance in patients, making the assessment less representative of their abilities in everyday situations. Indeed, upper limb movements have been shown to influence spatial attention performance [12,13], suggesting that the response mode itself may participate in the spatial processing of the task. Moreover, manual responses may recode auditory space relative to the acting limb or the response device [14], while ASA appears to rely on head-centered and oculocentric reference frames [15].

Such methodological challenges partly explain why, to our knowledge, no dedicated and validated tool is available for ASA assessment in clinical practice [4]. Thus, in this work, we developed a novel approach to assess ASA. This approach is based on recent advances in auditory virtual reality, eye tracking, and head-tracking technologies, which have created new opportunities for developing ecologically valid assessments. However, these technologies remain relatively uncommon in routine clinical practice and require the integration and synchronization of multiple hardware and software systems. Furthermore, for assessment tools to be adopted in clinical practice, they must demonstrate both adequate psychometric properties and a satisfactory user experience [16,17]. Indeed, technology-based assessments may be influenced by a range of technical and human factors that must be carefully evaluated to ensure the collection of reliable and interpretable physiological measures [18,19].

Thus, for the validation of our ASA assessment, we rely on the terminology and validity framework proposed in the *Standards for Educational and Psychological Testing* (2014), which defines validity as the extent to which evidence supports the interpretation of test scores for their intended use [20]. In this context, accumulating multiple sources of validity evidence is foremost when the assessment is intended for diagnostic purposes. Face validity and content validity constitute essential preliminary steps to ensure that the assessment appears to measure what it is intended to measure and that its components, including tasks and instructions, are appropriately aligned with the construct of interest [21-23]. Additional sources of evidence may further support whether the assessment elicits the expected cognitive processes, produces consistent scores, and provides accurate and fairly interpretable scores for all individuals. In addition to psychometric validity, usability, acceptance, and satisfaction are important considerations for identifying areas for improvement and facilitating implementation in clinical practice [18,24,25].

### Study Aims

This study aims to develop a new ASA assessment. As such, it addresses both the need for clinically applicable ASA assessment tools and the challenges associated with their implementation. Thus, phase 1 (completed) aimed to develop the first version of the ASA assessment. Phase 2 (ongoing) aimed primarily to evaluate the preliminary validity and secondarily to evaluate its user experience through 3 studies involving clinicians (study 1), healthy participants (study 2), and patients (study 3; planned subsequently). The present protocol covers phase 1 and part of phase 2 and contributes to the iterative refinement of the assessment within a broader 5-phase development process, illustrated in Figure 1.

**Figure 1.**
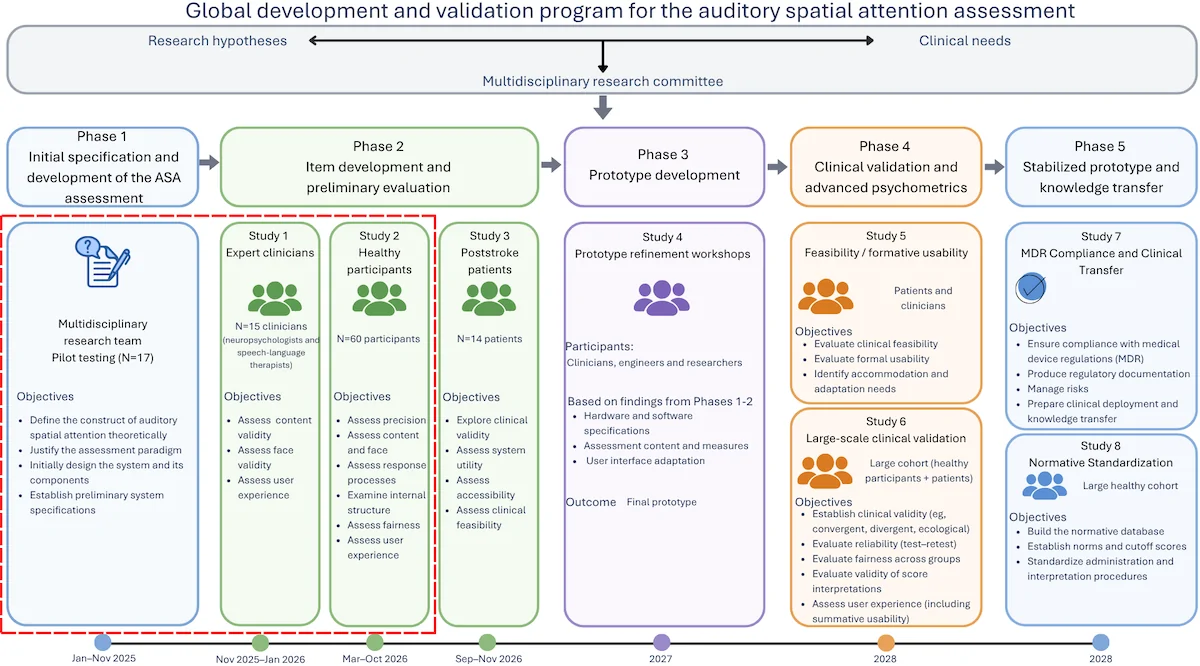
Overview of the different phases and studies involved in the development and validation of the auditory spatial attention assessment. The red dashed box indicates the scope of the present protocol paper. The timeline is indicative and reflects the anticipated progression of the research program. ASA: auditory spatial attention; MDR: medical device regulations.

On the basis of the gap between experimental developments and clinical practice, and the importance of developing a clinically transferable approach using eye-tracking response modes and auditory virtual reality, the assessment development process is based on the *Universal Design* described in *Standards for Educational and Psychological Testing* (2014). This type of design consists in developing an assessment with as few access barriers as possible, making it usable by the widest possible range of participants regardless of their individual characteristics. That is why we first involve expert clinicians and healthy participants before subsequently evaluating the assessment in different patient populations.

## Methods

### Ethical Considerations

Phase 1 involved preliminary pilot work with healthy participants and received ethical approval from the Institutional Research Ethics Committee of the University of Caen (reference number 2024092711253400000160000391). They received a detailed information sheet and an informed consent form outlining the study objectives, procedures, and data handling. In addition to these study participants, members of the participatory research team, including clinicians, engineers, and researchers, contributed to the iterative codevelopment of the assessment. These contributions were part of the participatory design process rather than the research study itself. Therefore, these individuals were not considered study participants; their feedback was not collected or analyzed as research data, and written informed consent was not required.

Phase 2, including study 1 with a group of expert clinicians participants and study 2 with healthy participants, received ethical approval from the Institutional Research Ethics Committee of the University of Caen (reference number 2025060606545200000310000391). Expert clinicians and healthy participants will receive a detailed information sheet and an informed consent form outlining the study objectives, procedures, and data handling. Written informed consent will be obtained from each participant on the day of data collection. All collected data will be anonymized before analysis, and participants will be informed that they may withdraw from the study at any time without any consequence.

### Phase 1: New ASA Assessment Approach Development (Completed)

#### Objectives and Overview

The objective of phase 1 was to develop the initial version of the ASA assessment through a collaborative process involving a multidisciplinary team of clinicians, engineers, and researchers. First, several team meetings were conducted, during which 10 clinicians were invited to discover and, if they wished, interact with a preliminary version of the assessment. Their feedback, discussed within the participatory research team, led to several modifications of the ASA assessment. The resulting version was then discussed with 3 additional clinicians, leading to further adjustments. Finally, the approach was tested with 17 healthy participants, resulting in a reduction in the number of silhouettes and sound-source positions and adding a control condition without visual inferences. This iterative development process resulted in the version of the ASA assessment described in the remainder of this section, which constitutes the basis for the preliminary validation presented in phase 2.

#### System Design Rationale

##### Localization Task

Our new approach was built on a sound localization task, a paradigm commonly used to engage ASA networks [26]. However, the aim was not to assess localization accuracy per se. Rather, sound localization was used as a means of engaging auditory spatial-attention mechanisms. In the developed ASA assessment, sound positions were selected to engage broad auditory spatial-attention regions while reducing the risk of overlap between adjacent spatial categories. This choice was supported by evidence suggesting that ASA operates according to a spatial attentional gradient rather than strictly isolated locations. Orienting attention toward a given sound position has been shown to enhance the processing of nearby locations as well [27], suggesting that ASA is organized around spatial regions. In addition, increasing the number of spatial locations (ie, not only left or right) has been associated with stronger engagement of auditory spatial-attention networks [26], making the task less reliant on simple perceptual discrimination and more dependent on auditory spatial-attention processes.

Accordingly, the auditory environment was designed to provide sufficiently salient spatial cues to allow listeners to perceive sounds as originating from different regions of space. To achieve this, several components were designed to facilitate sound localization and place participants in optimal perceptual conditions. The goal was not to evaluate fine-grained localization abilities but rather to ensure that spatial information can be reliably perceived so that performance primarily reflects higher-level auditory spatial-attention processes.

##### Immersive and Interactive Virtual Auditory Environment

To deliver the auditory stimuli of the assessment, a virtual auditory environment was developed. Virtual auditory environments are computer-generated environments that can provide immersive and interactive auditory experiences [28-30]. Such environments create rich auditory scenes containing multiple spatial regions that may support the deployment of ASA.

###### Immersion Through Binaural Rendering

Immersion was achieved through binaural rendering, which has been shown to support the construction of spatial representations and the engagement of auditory spatial-attention networks [6]. Binaural rendering is considered one of the most natural approaches for simulating real-world auditory environments [31] and can be delivered through standard headphones. Compared with loudspeakers, headphones are quick and easy to place on participants, independent of room acoustics and of the participant’s position, and less expensive, key advantages for clinical applications [32]. Additionally, binaural rendering recreates a natural listening experience by delivering to each ear the signal that would naturally occur in a real sound field, thereby preserving localization cues while allowing precise control over sound presentation [33].

This natural listening experience is possible thanks to head-related transfer functions (HRTFs), mathematical filters that model how the body (head, torso, and outer ears) modifies incoming sound waves. For each sound position, the HRTF computes the expected signal at the eardrum, relative to these body modifications, allowing us to recreate in headphones the natural differences between the 2 ears [34]. This process makes the simulation both realistic [34] and suitable for engaging ASA networks [6] by capturing three key cues required for accurate sound localization [34] (Figure 2):

1. Interaural time difference (ITD): Sound reaches the 2 ears with a small difference in time of arrival that depends on the horizontal angle of incidence (azimuth) [35]. For example, a sound coming from directly in front (0°) or behind (180°) yields an ITD ≈0 milliseconds, whereas a sound arriving from 90° on the right produces a near-maximal ITD of about 0.6‐0.8 milliseconds. This arises from the anatomical placement of the ears on opposite sides of the head. This cue is used for horizontal (azimuthal) localization.
2. Interaural level difference (ILD): Amplitude (level) at the 2 ears differs owing to head shadowing [35]. Attenuation is maximal for near-perpendicular incidence (≈90° azimuth), preferentially reducing the signal at the ear contralateral to the source. As with ITD, ILD primarily supports azimuthal (horizontal) localization.
3. Spectral cues: Reflections and diffractions caused by the head, shoulders, chest, and outer-ear cavities modify the incoming sound wave. These phenomena create direction-dependent spectral changes that vary with the angle of incidence. The brain learns to associate these timbral alterations with specific directions, which also enables elevation (vertical) localization. These spectral cues complement the information provided by ITD and ILD and also contribute to azimuthal (horizontal) localization [36].

**Figure 2.**
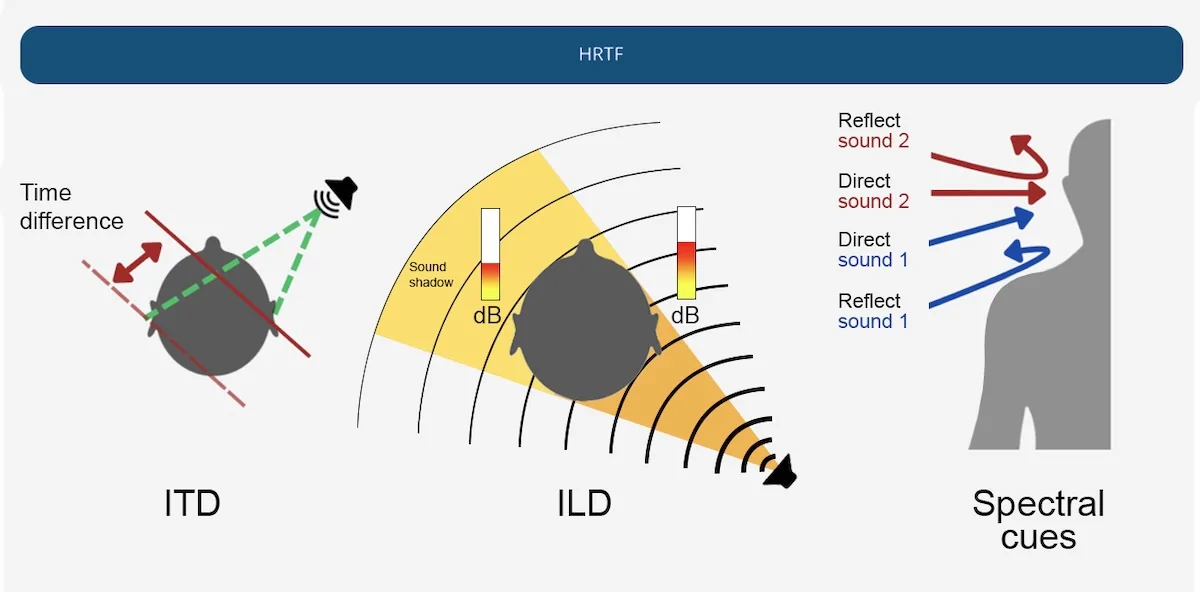
Illustration of sound localization cues contained in the HRTF. HRTF: head-related transfer function; ILD: interaural level difference; ITD: interaural time difference; Spectral cues: modifications of the sound wave by the head, shoulders, chest, and outer-ear cavities.

###### Interaction Through Head Movement Adaptation

To achieve interaction and further support spatial sound perception, the auditory virtual environment was dynamically adapted to listeners’ head movements. Head movements naturally contribute to spatial hearing [37] and allow the system to continuously update spatial cues through the dynamic adaptation of HRTFs. Indeed, because the sound signal is modified by individual anatomical characteristics, HRTFs differ across individuals, but there is no straightforward measurement process to individualize HRTFs [34,38]. This leads us to use generic HRTFs that commonly result in front-back confusions in sound localization [39,40] and poor externalization [41], potentially disorienting participants in our assessment. Head movement–adaptive environments compensate for these issues [41] even for small head rotations (<4°) [42]. Also, previous studies have shown that the latency between head motion and auditory update should remain below approximately 25 milliseconds to preserve a stable spatial perception [43]. This highlights the importance of using high-quality hardware to take full advantage of head movements.

### Eye Tracking–Based Response Mode

In classical visual spatial attention tasks, performance is often characterized by both the extent of the explored visual field and the attentional cost; it is therefore common to observe patients who complete the task with a normal extent of visual field exploration but elevated attentional cost, suggesting impaired cognitive function.

The extent of the explored attentional field is reflected by gaze orientation and fixation patterns [44], and a similar relationship may exist in ASA. Indeed, studies have shown that eye movements are naturally involved in auditory spatial orienting. More broadly, ASA tasks recruit visual cortical areas [7], even in congenitally blind individuals for whom eye movements are not behaviorally relevant during auditory tasks [11], suggesting that gaze orientation constitutes a natural response modality for ASA. In addition, gaze orientation has been shown to facilitate sound localization [45]. As sound localization is used in the present assessment as a means of engaging ASA, gaze-based responses may help support task performance without introducing additional response-related constraints. Finally, eye-tracking paradigms offer an ecological and intuitive response modality for ASA assessment. Indeed, eye movement–based localization tasks are rapid, accurate, and associated with relatively low cognitive and motor demands [46].

Simultaneously with gaze orientation recording, the eye-tracking system also enables the assessment of attentional cost through variations in pupil diameter. Pupillometry therefore provides a complementary physiological marker of attentional load, as pupil size increases with increasing cognitive demand. It has been shown relevant for measuring (1) the impact of auditory attention switching [47] and (2) the cost of divided auditory attention [48,49].

### Benefits of a Visual Environment for Sound Localization

Although our primary aim was to assess ASA, a visual environment was added to the assessment for two main reasons: (1) to provide a stable and shared spatial framework to help participants associate target sounds with attentional spatial areas, and (2) to provide a visual fixation point that stabilizes gaze, reduces interfering eye movements, and thereby improves data quality.

The visual environment was intended to provide spatial support for the attentional areas toward which ASA is expected to be oriented by seeing representation of the sound sources. Indeed, in a sound localization task, the presence of a visible loudspeaker improves participants’ performance, demonstrating the facilitative effect of visual cues [50]. This also increases ecological validity, as most everyday sounds are accompanied by visual information, although some situations rely exclusively on auditory cues.

Furthermore, the interpretation of eye-tracking data can be affected by experimental device and participant behavior [51]. Without a visual marker, participants may visually explore anywhere in space in search of the sound source, which compromises gaze stability and fixation accuracy, thereby making the interpretation of eye-tracking data more difficult [52]. These difficulties can also be exacerbated by alterations in attentional capacity, as in patients [52]. Therefore, providing visual support is essential to constrain gaze behavior and ensure that recorded measures reflect attentional processes rather than methodological bias of gaze acquisition.

### Head-Tracking Response Mode

To verify that the assessment configuration does not introduce supplementary cognitive processes, an additional response mode based on head tracking was included. Indeed, head movements have been associated with ASA and listening-orientation behaviors in natural environments [53,54]. Additionally, the head-tracking response mode was selected because it did not require visual reference points for participants to indicate the region of space toward which they directed their ASA. While this may provide a relevant response mode, it is also associated with greater interindividual variability due to individual differences in head orientation responses to auditory spatial locations and may not be suitable for some clinical populations (eg, poststroke patients with restricted head movements). Therefore, head tracking was intended to serve as a complementary outcome measure, in order to provide additional evidence regarding whether performance was primarily driven by auditory spatial attention processes or by other task-related processes.

### ASA Assessment

#### System Overview

A sound localization task designed to assess ASA was developed using the Unity game engine and clinically compatible hardware. Figure 3 shows an overview of the system components, implemented by coupling several software (Unity, Max/MSP, IRCAM SPAT) and hardware tools (Supperware and Tobii).

**Figure 3.**
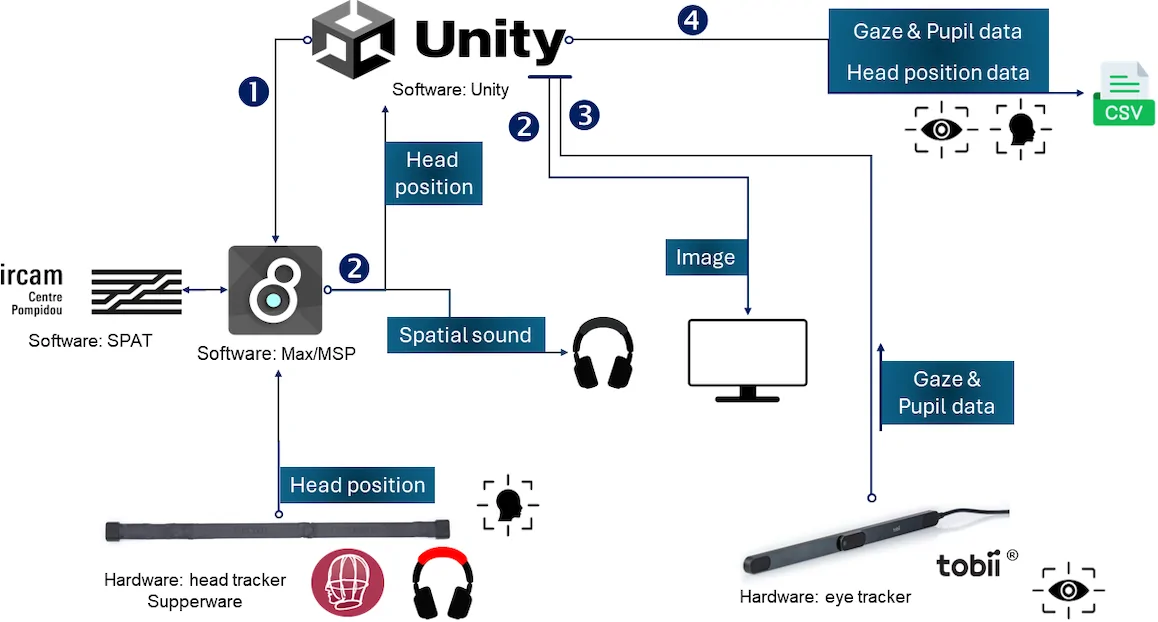
Experimental setup used for acquiring gaze, pupil size, and head position data. Numbers (1-4) correspond to the chronological steps of the procedure. All product names, logos, and brands are property of their respective owners. All trademarks are acknowledged and used for identification purposes only.

Immersive auditory virtual environments consist of target sounds (vocal stimuli) and a background sound (coffee shop ambiance), all specifically recorded for this study. The target sound bank includes 20 French sentences with a mean duration of 1.8 seconds (maximum: 2.1 seconds), all following the same structure (eg, “Good morning, my name is Marie, nice to meet you”), with 10 sentences spoken by different women speakers and 10 by different men speakers. To standardize the stimuli, silence was added at the end of each sentence, resulting in a total duration of 3.1 seconds, which also allowed the system enough time to load the next sound. Each sentence is spatialized in front of the participant’s head at 1 of 5 fixed locations to engage multiple auditory spatial attention areas: 90° left (L90), 45° left (L45), 0° center (C00), 45° right (R45), and 90° right (R90). Unity sends instructions to Max/MSP regarding the target sound characteristics (sentence, speaker gender, and background sound condition) and spatial location for each trial. Max/MSP then performs binaural rendering (see Multimedia Appendix 1 for detailed auditory environment specifications), and the sounds are delivered through Beyerdynamic DT 770 Pro headphones.

In order to provide *interaction between head movement and the auditory virtual environment*, a Supperware external inertial head-tracking system is incorporated into the headphones, with a manufacturer-reported theoretical latency of less than 20 milliseconds (see Multimedia Appendix 1 for details of the theoretical end-to-end latency). Indeed, most commercially available headphones do not include integrated head-tracking, or, when they do, the resulting data are not accessible for analysis. Thus, the selected head-tracking system continuously sends head position data to Max/MSP via musical instrument digital interface over USB. Max/MSP then uses the Spat5 real-time audio processor from the IRCAM SPAT library to spatialize sounds according to head position, enabling dynamic binaural rendering (ie, real-time HRTF adaptation) [55].

*Gaze orientation and pupil-size variation measurements* are enabled using a Tobii Eye Tracker 5L (Tobii AB [56]), a contactless screen-mounted infrared eye tracker operated in high-speed mode at 120 Hz. The device was selected because it is suitable for clinical use due to its ease of use and compliance with hygiene standards. The eye tracker continuously sends gaze position and pupil diameter data to Unity. The choice of a screen-mounted eye tracker was also motivated by the limitations associated with alternative technologies such as head-mounted displays (HMDs) and eye-tracking glasses. HMDs present several limitations, including cybersickness, which may be exacerbated by hardware parameters and/or individual factors [57], medical contraindications (eg, epilepsy, high myopia, and postural instability), other adverse effects (eg, headaches or even delusional episodes) [58], and practical implementation constraints (eg, time-consuming setup procedures and the need for technical support) [59]. Eye-tracking glasses also have limitations, including difficulties in accurately quantifying viewed regions because of head movements, sensitivity to facial movements (eg, emotional expressions), and slippage of the glasses during recording [60].

*To support gaze-based responses and ASA, 2 visual environments* are displayed alternatively: 1 displaying potential sound source positions and 1 showing a fixation cross. Potential sound source positions (Figure 4) are represented by identical neutral, black, and static silhouettes (same shape and size), with woman silhouettes matching woman speakers’ voices (Figure 4A) and man silhouettes matching man speakers’ voices (Figure 4B), presented on a gray background to minimize visual distraction and ensure that recorded responses primarily reflect ASA. A schematic representation of the participant (head with headphones) is placed at the bottom center of the screen to provide a reference for their position within the auditory spatial environment. Before each trial, a fixation cross is displayed at the bottom center of the screen to standardize the initial gaze position and provide a cue for the onset of the next sound [15]. The visual environment is controlled by Unity and displayed for 3.1 seconds during each target sound in the eye-tracking response-mode blocks.

**Figure 4.**
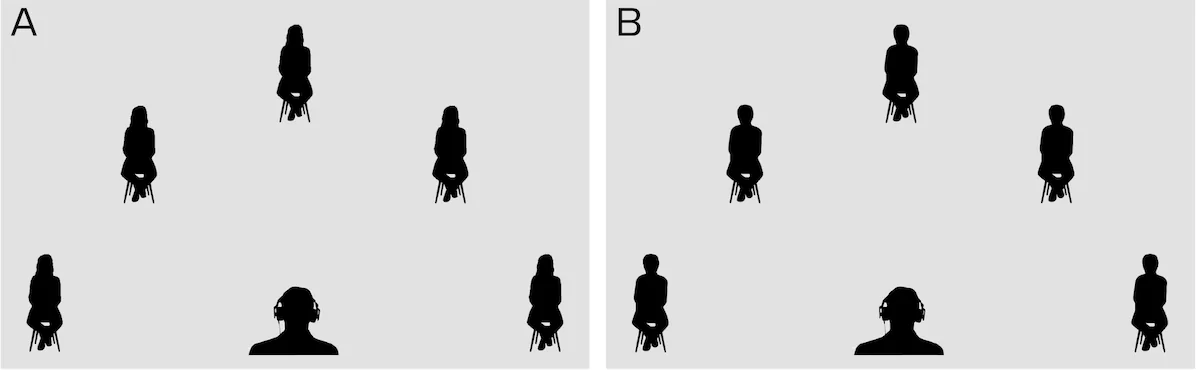
Images used during listening to the vocal sound in the eye-tracking or pupillometry condition only. (A): woman silhouette. (B): man silhouette. Not displayed during head orientation responses.

Finally, *head position* measurement is enabled through the transmission of data recorded by the Supperware head tracker to Unity via Max/MSP. During these phases, participants receive verbal instructions generated using artificial intelligence–assisted speech synthesis (ElevenLabs). At the end of each block, Unity exports a comma-separated values file sampled at 120 Hz, containing time-stamped information on the presented sounds, head position, gaze position, and pupil size (see the “ASA Assessment Tasks” section for a detailed description of the block structure).

#### ASA Assessment Tasks

The tasks consist of 5 phases (Figure 5): 2 eye-tracking phases (phases 1 and 2 in Figure 5) and 3 head-tracking phases (phases 3‐5 in Figure 5).

**Figure 5.**
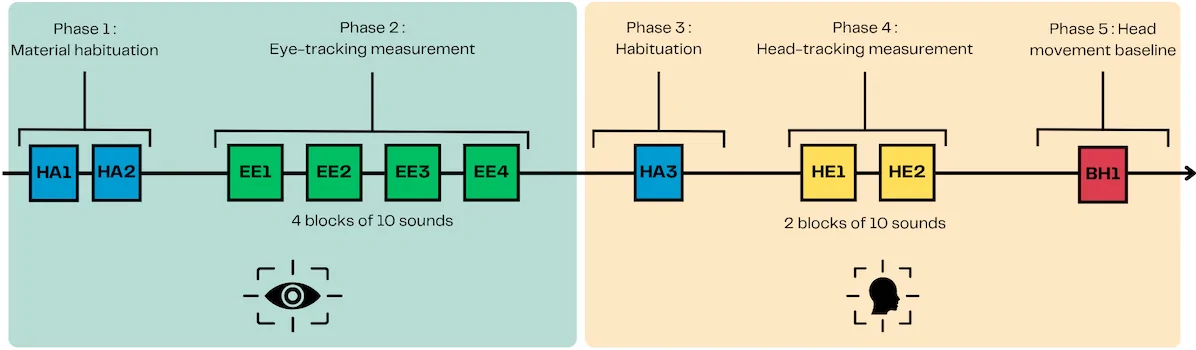
Recordings consist of multiple tasks divided into 5 main phases. Phase 1 (material habituation): HA1 (HAbituation to the visual environment) and HA2 (HAbituation to the audiovisual environment). Phase 2 (eye-tracking experimental task): EE1 to EE4. Phase 3 (habituation): HA3 (equipment HAbituation). Phase 4 (head-tracking experimental task): HE1 and HE2. Phase 5 (head movement baseline): BH1.

To perform the task, participants are seated 60 cm from a computer monitor (HP E24i G4, 1920×1200 pixels, 51.84×32.4 cm) connected to the control computer (ROG Strix G531G, Windows 10), which enables the experimenter to regulate the timing and progression of the different assessment phases. The control computer and experimenter are located on the other side of a curtain, which serves to attenuate room light and minimize the potential influence of the experimenter’s presence, particularly when participants have their eyes closed. Before beginning the ASA assessment, eye-tracking is calibrated using Tobii’s native 6-point procedure, and head tracking is calibrated using Supperware’s native procedure.

The eye-tracking phases (phases 1‐2) are designed to assess attentional orienting through gaze direction and attentional cost through pupil-size variations. Participants sit facing the computer screen with their eyes open and are instructed to avoid large head movements. Two habituation blocks are first provided. The first block (HA1) familiarizes participants with the visual environment. The second block (HA2) familiarizes them with the complete trial sequence, including the fixation cross, spatialized target sounds presented from locations corresponding to the 5 silhouettes, and the associated visual display. The rationale for using habituation rather than training is detailed in Multimedia Appendix 2. Participants then complete 4 experimental blocks of 10 trials each (EE1-EE4), during which target sounds are presented from 1 of the 5 predefined spatial locations associated with the silhouettes, in randomized order. Figure 6 illustrates the timeline of a single trial. Following fixation on the fixation cross, participants are instructed to identify the silhouette corresponding to the region of space from which they perceive the target sound to originate and to maintain their gaze on this silhouette until the visual display disappears.

**Figure 6.**
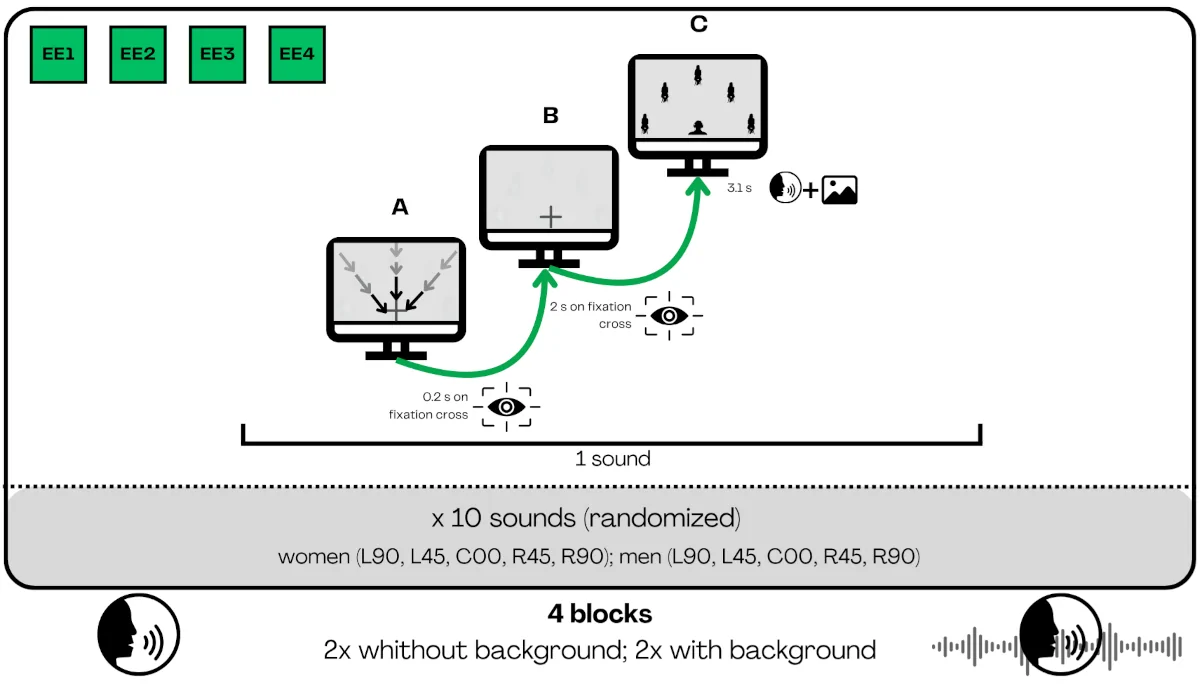
Representation of the experimental phase based on eye-tracking measurement (EE1 to EE4). The screen successively displays (A) the fixation cross with dynamic arrows, (B) the fixation cross alone, and (C) the vocal sounds together with the silhouettes image that participants are required to fixate.

The head-tracking phases (phases 3‐5) are designed to assess attentional orienting through head direction. Participants remain seated facing the computer screen, as the fixation cross is used to recenter head position between trials but perform the localization task with their eyes closed. A habituation phase familiarizes participants with the complete trial sequence, including the fixation cross, auditory instructions indicating when to close their eyes, spatialized target sounds, and auditory instructions indicating when to open their eyes and return to the fixation cross. Participants then complete 2 experimental blocks of 10 trials each (HE1 to HE2), during which target sounds are presented from randomized spatial locations. They are instructed to orient their head toward the perceived target sound source and maintain this position until the sound ends. Figure 7 illustrates the timeline of a single trial. In the final block (BH1), participants are instructed to turn their head twice as far as possible to the right and twice as far as possible to the left to measure their maximum head orientation amplitude.

**Figure 7.**
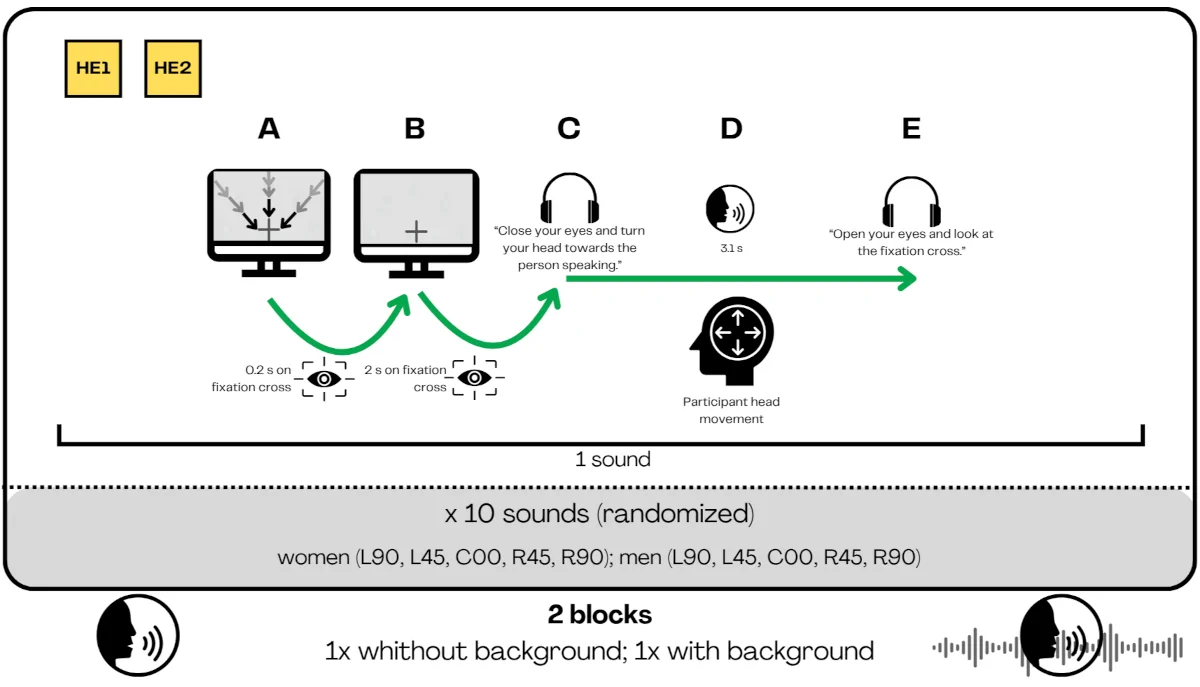
Representation of the experimental head-tracking phases (HE1 and HE2). The task successively presents (A) the fixation cross with dynamic arrows, (B) the fixation cross alone, (C) the audio instruction to close the eyes and orient the head toward the sound, (D) the target sound toward which participants must orient, and (E) the audio instruction to open the eyes and return gaze to the cross.

In both assessment modalities, target sounds are presented from 5 spatial positions (L90, L45, C00, R45, and R90). Target sounds are presented in all experimental blocks, with half of the blocks including background sound and the other half presented without background sound. Within each 10-trial block, each spatial position is presented twice, once with a woman voice and once with a man voice, in randomized order. Each trial has a fixed duration of 3.1 seconds. Detailed procedural descriptions and schematic representations of each phase are provided in Multimedia Appendix 2.

### Phase 2 (Study 1): Preliminary Validity and User Experience Evaluation With Expert Clinicians (Ongoing)

#### Objectives and Overview

The first objective is to assess content and face validity to determine whether the proposed assessment contains components that are relevant, clear, comprehensive, and appropriately aligned with the construct it is intended to measure, that is, ASA. A second objective is to evaluate user experience, including usability, satisfaction, and acceptance. ASA assessment preliminary validation follows the steps described in the *Standards for Educational and Psychological Test* (2014) and the COSMIN methodology [20,23]. We will use both qualitative and quantitative methods.

#### Trial Population and Recruitment

The target sample size is 15 expert clinicians, as this number is considered sufficient for evaluating content and face validity, which constitute the main objectives of study 1 [61]. Expert clinicians will be required to meet the following inclusion criteria: being a speech therapist or neuropsychologist, having at least 1 year of clinical practice, and having a minimum of 6 months of experience in attention assessment. They will not be eligible if they self-report any of the following exclusion criteria: neurological, neurodegenerative, or psychiatric disorders, oculomotor impairments, visual or auditory extinction, or hearing impairment. Hearing impairment will also be assessed objectively using AudioSchool pure-tone audiometry (mean hearing threshold across the standard test frequencies of 500, 1000, 2000, and 4000 Hz) and the Höra speech-in-noise test [62]. Participants with a mean hearing threshold >25 dB HL or a Höra score <50% will be excluded. As expert clinicians will be required to perform the assessment tasks under evaluation, these exclusion criteria will be applied to minimize the potential influence of sensory, neurological, or oculomotor impairments on validity and user-experience evaluations.

A snowball sampling strategy will be used to recruit expert clinicians. While non–random sampling approaches may limit the generalizability of findings, they are considered appropriate for exploratory phases of assessment development [63]. Expert clinicians will initially be contacted through professional networks and will be invited to share the study invitation with colleagues meeting the eligibility criteria. Clinicians interested in participating will be asked to contact the research team directly.

#### Data Collection

Data collection will be organized in five steps: (1) pretests, (2) experimental tasks, (3) posttests, (4) postsession, and (5) feedback session. Expert clinicians will participate in 3 sessions: 2 laboratory sessions of approximately 1.5 hours (1 covering steps 1‐3 and 1 covering step 5) and 1 remote session covering step 4.

##### Step 1: Pretest

Once written informed consent has been obtained, expert clinicians will complete questionnaires including sociodemographic information, musical experience (questionnaire developed by our team and inspired by items from the MUSE questionnaire; [64]), and manual laterality (Edinburgh Inventory [62]). The experimenter will also administer additional assessments: tonal auditory perception (AudioSchool audiometer), speech-in-noise perception (Höra application, validated in French [65]), and ocular dominance (near hole-in-the-card test [66]).

##### Step 2: Experimental ASA Assessment

Expert clinicians will be seated at a desk in a quiet and dimly lit room in front of a computer monitor. After completion of the eye-tracking and head-tracking calibration procedures, participants will complete the 5 phases of the ASA assessment, during which eye-tracking and head-tracking data will be acquired (see the “ASA Assessment Tasks” section). During assessment, different measures will be collected: gaze position, pupil size, and head position.

##### Step 3: Posttest

After completing all tasks of the ASA assessment, expert clinicians will be asked to complete a questionnaire on user experience (satisfaction) and face validity (English version is available in Multimedia Appendix 3).

##### Step 4: Postsession

Expert clinicians will receive a questionnaire by email to evaluate content validity, usability, and intention to use. A brief reminder of the tasks will be provided before completion of the questionnaire. The use of a self-administered questionnaire completed outside the laboratory setting is considered appropriate, as expert clinicians will have returned to their clinical environment and may be better able to envision real-world use while having sufficient time to reflect on each component (English version is available in Multimedia Appendix 3).

##### Step 5: Feedback Session

Once the results have been analyzed, a feedback session will be organized to present the findings, with particular emphasis on the content-validity results. The objective of this session will be to identify consensual solutions for assessment components that did not reach the predefined validity criteria. During the session, the item-level content validity index (I-CVI) will be recalculated in real time after each proposed modification to determine whether the predefined consensus threshold has been reached. If consensus is not reached by the end of the session and some assessment components remain unvalidated, modifications will be implemented and presented during up to 2 additional feedback sessions. This iterative process is commonly used in consensus-building methodologies for which 2-3 rounds are often sufficient to achieve agreement among participants [67].

### Outcomes Measures

Different indicators will be used to assess preliminary validation and user experience. For clarity, they are presented in Table 1.

**Table 1.** Overview of outcomes, descriptions, and indicator.

Objective	Step collection	Description	Outcomes measures	Instrument
Face validity	3	Whether the assessment appears to measure ASA^a^	Yes/no question with justification	Study-specific questionnaire
Content validity (comprehensiveness)	4	Whether the assessment includes all relevant components for assessing ASA	Yes/no question with justification	Study-specific questionnaire
Content validity (relevance and clarity)	4	Whether the assessment includes components that are relevant and clear for assessing ASA	5-point Likert scales	Study-specific questionnaire
User experience— Usability	4	Overall usability of the assessment	SUS^b^, 5-point Likert scale	Validated questionnaire (French SUS; Gronier and Baudet [68])
User experience— Satisfaction	3	Experience with the assessment, including motivation, enjoyment, perceived sound localization, and overall appreciation	Four open-ended questions and four 5-point Likert scale	Study-specific questionnaire
User experience— Intention to use	4	Perceived usefulness of the assessment and intention to adopt it in clinical practice	Four 5-point Likert items (2 perceived usefulness; 2 behavioral intention)	Study-specific questionnaire inspired by TAM^c^ (Davis [69]) and UTAUT^d^ (Venkatesh et al [70])

a ASA: auditory spatial attention.

b SUS: System Usability Scale.

c TAM: Technology Acceptance Model.

d UTAUT: Unified Theory of Acceptance and Use of Technology.

### Statistical Analyses

All statistical analyses will be conducted using RStudio (version 2026.07.0+139; Posit Software, PBC). Statistical significance will be set at *P*<.05. Participants with missing data will be excluded only from analyses requiring the missing variables, while remaining eligible for all other analyses. In the case of missing data, the number of participants included in each analysis will be reported.

#### Validity

*Face validity and content validity of comprehensiveness* will be analyzed through the percentage of clinician experts expressing favorable or unfavorable opinions. In addition, a thematic coding of the justifications provided will be conducted using a general inductive approach combined with a deductive method [71]. Agreement rates of at least 75% will be considered supportive of consensus, in line with commonly used approaches such as Delphi studies [67]. *Content validity of relevance and clarity* will be established using the I-CVI [72], calculated as follows: (number of experts rating the item as 3 or 4)/(total number of experts). To be considered relevant or clear, the threshold is set at 0.78, which is commonly used in the literature [71,73]. Components with scores below this threshold will be discussed during the feedback session. See Table 2 for a detailed overview of the links between the study hypotheses and the planned validity analyses.

**Table 2.** Planned validity hypotheses, outcome measures, statistical analyses, and criteria supporting validity of study 1.

Validation domain	Statistical hypothesis	Statistical analysis	Result supporting validity
Face validity	A majority of clinicians will judge whether the proposed tasks adequately engage auditory spatial attention processes.	Descriptive statistics (%)	≥75% agreement
Content validity (comprehensiveness)	Clinicians will judge the assessment content to be sufficiently comprehensive.	Descriptive statistics (%)	≥75% agreement
Content validity (relevance and clarity)	Assessment components will reach predefined content validity thresholds for relevance and clarity.	I-CVI^a^ calculation	All items: I-CVI ≥0.78

a I-CVI: item-level content validity index.

#### User Experience

*Usability* scores on the System Usability Scale will be analyzed following the standard scoring procedure [68] and interpreted using the threshold scores proposed by Bangor et al [74]. Qualitative feedback from participants will also be considered, as usability issues may be identified even when overall System Usability Scale scores indicate low usability. *Satisfaction *will be analyzed through the median scores of the satisfaction items, which will be compared against the neutral value (3) using 1-sided 1-sample Wilcoxon signed-rank tests. Qualitative responses will be analyzed thematically. *Intention to use* will be analyzed through the median scores of each item, which will be compared against the neutral value (3) using 1-sided 1-sample Wilcoxon signed-rank tests [75]. See Table 3 for a detailed overview of the links between the study hypotheses and the planned user experience analyses.

**Table 3.** Planned validity hypotheses, outcome measures, statistical analyses, and criteria supporting no modification of study 1.

User experience domain	Statistical hypothesis	Statistical analysis	Result supporting no modification
User experience—Usability	Clinicians are expected to report acceptable usability, as this is a first assessment version.	SUS^a^ score versus interpretative thresholds	SUS≥68 *and *no major usability issues identified
User experience—Satisfaction	Clinicians are expected to report moderate levels of satisfaction, as this is a first assessment version.	Thematic analysis + 1-sided 1-sample Wilcoxon signed-rank test	Predominantly positive themes and *P*<.05
User experience—Intention to use	Clinicians are expected to perceive the assessment as useful and report a positive intention to use it.	Descriptive statistics + 1-sided 1-sample Wilcoxon signed-rank test	*P*<.05

a SUS: System Usability Scale.

Progression to the next phase involving healthy participants will depend on predefined face validity and comprehensiveness criterion of at least 75% agreement among expert clinicians, as well as achieving the predefined content validity threshold (I-CVI ≥0.78) for all components evaluated in terms of relevance and clarity. If these criteria are not met, additional iterations may be conducted (see Step 5: Feedback Session section). In line with the iterative nature of the validation process, the assessment will be further refined, if necessary, based on findings from the validity and user experience evaluations before initiating the next phase with healthy participants.

### Phase 2 (Study 2): Preliminary Validity and User Experience Evaluation With Healthy Participants

#### Objectives

The first objective is to gather different sources of validity evidence. This includes evidence based on (1) reliability and precision by evaluating the accuracy of localization responses relative to sound-source locations; (2) face and content validity; (3) response processes by examining whether eye tracking, head tracking, and pupillometric responses reflect ASA as intended; and (4) internal structure by determining whether performance patterns across spatial positions and background noise conditions are consistent with the theoretical structure underlying the assessment. The second objective is to evaluate fairness by determining whether individual characteristics unrelated to ASA artificially influence assessment performance. The third objective is to evaluate user experience, including usability, satisfaction, and sense of presence as an indicator of ecological relevance.

#### Trial Population and Recruitment

The target analyzable sample size is 80 healthy participants, equally distributed across 2 age groups (18‐40 years and 60‐80 years). The sample size was determined based on the requirements of the generalized linear mixed-effects model, which requires the greatest sample size among the exploratory validation analyses (primary objective of the study). Simulation-based power analyses were conducted in R using the *simr* package [76]. Assuming a 2-sided significance level of .05 and a statistical power of 80%, the simulations indicated that a total sample of 80 participants provides at least 80% power to detect medium-sized effects of the primary predictors (sound position and background noise). Other planned quantitative analyses require an equal or smaller sample size. Participants will be eligible if they are healthy adults within one of these age ranges and do not meet any of the exclusion criteria described for study 1.

A convenience sampling strategy will be used to recruit participants for the same reasons as those described for study 1 [63]. Recruitment announcements will be distributed through social networks and public spaces. Individuals interested in participating will be invited to contact the research team by email or telephone to obtain additional information about the study and to verify their eligibility. Eligible participants will then be provided with an information sheet. If they remain interested after reviewing the study information, they will be invited to notify the research team. A member of the research team will then contact eligible participants to schedule a data collection session, which will take place after written informed consent has been obtained.

#### Data Collection

Data collection will follow procedures similar to those used in the first 3 steps of study 1, as steps 4 and 5 will not be included. Therefore, only the differences between the 2 protocols are presented in the following text. Participants will engage in 1 session in the laboratory for approximately 1 hour.

1. *Step 1* (*pretest*): This step will be the same as that of study 1.
2. *Step 2* (*experimental ASA assessment*): Unlike study 1, the order of the 2 tracking modalities (eye-tracking first or head-tracking first) will be counterbalanced across participants to allow the evaluation of response process validity.
3. *Step 3* (*posttest*): After completing all tasks of the ASA assessment, participants will be asked to complete a questionnaire (English version is available in Multimedia Appendix 4) on face validity, content validity, user experience (usability and satisfaction), and sense of presence.

#### Outcomes Measures

Several new indicators will be used in this study to assess preliminary validation and user experience. For clarity, they are presented in Table 4.

**Table 4.** Overview of outcomes, descriptions, and indicators.

Objective	Description	Outcomes measures	Instrument
Reliability or precision	Accuracy of eye- and head-tracking responses relative to the sound source location.	Gaze performance, head performance, and sound source position	ASA^a^ assessment task
Face and content validity	Participants’ perception that the assessment appropriately engages auditory spatial attention and uses appropriate content	Five closed-ended yes/no questions Six 5-point Likert scale items	Study-specific questionnaire
Validity based on response processes	Consistency of participants’ reported strategies with spatial attention engagement	Two open-ended questions	Study-specific question
Validity based on response processes	Consistency between eye- and head-tracking performances during sound localization	Gaze performance and head performance	ASA assessment tasks
Validity based on response processes	Influence of tracking order on ASA performance and response patterns	Composite performance score (eye | head)	ASA assessment tasks
Validity based on response processes	Consistency between physiological responses and perceived task difficulty	Mean and peak pupil size, 2 open-ended questions	ASA assessment tasks and study-specific questions
Validity based on internal structure	Effect of sound-source position and background sound on performance	Measures: Gaze performance and head performance Variables: sound position and background sound (yes/no)	ASA assessment tasks
Validity based on internal structure	Effect of sound-source position and background sound on attentional load	Measures: Pupil size (mean and peak) Variables: sound position and background sound (yes/no)	ASA assessment tasks
Fairness	Whether assessment performance is influenced by participant characteristics unrelated to auditory spatial attention	Age group, hearing score, and speech-in-noise score	Sociodemographic questionnaire, audiometry, and ASA assessment tasks
User experience— Usability	Participants’ perception of the overall usability of the assessment	SUS^b^; 5-point Likert scales	Validated questionnaire (French SUS [68])
User experience— Satisfaction	Participants’ perceptions and experiences of the assessment, including motivation, enjoyment, perceived sound localization, and overall appreciation	Twelve 5-point Likert scales and 3 closed-ended questions	Study-specific questionnaire
User experience— Sense of presence	Participants’ perception of behaving as if the testing situation were real	Eleven 5-point Likert scales	Study-specific questionnaire

a ASA: auditory spatial attention.

b SUS: System Usability Scale.

To determine eye-tracking and head-tracking performance, the proportion of correct final fixations or head positions relative to the total number of trials will be calculated separately for each sound position and background sound condition. For eye tracking, correct final fixations will be analyzed relative to the predefined regions of interest (ROIs). For head tracking, correct final head positions will be analyzed relative to predefined angular areas, with the central area corresponding to ±2.5° around 0°, values below −2.5° classified as left, and values above +2.5° classified as right. A composite score will also be created by calculating the total number of correct responses obtained from eye tracking and head tracking.

Hearing ability will be assessed using a hearing score based on pure-tone audiometry with the AudioSchool audiometer (mean pure-tone threshold across both ears, expressed in dB HL) and a speech-in-noise score obtained with the Höra application (score out of 100).

#### Eye-Tracking and Head-Tracking Measures

##### Overview

The developed methodology will enable fully automated processing of eye- and head-tracking data, thereby eliminating the labor-intensive manual steps usually required, such as selecting candidate fixations or calculating pupil size. This automation will be particularly relevant for eye-tracking data and will offer several advantages: it will remove the risk of analyst-induced bias [77], ensure optimal reproducibility by making results independent of the operator, and substantially reduce analysis time. In addition, the pipeline will require no advanced technical expertise, which will enhance its transferability to clinical settings where time and resources are often limited. The pipeline will include (1) fixation detection, (2) pupil diameter calculation, and (3) determining the head position (Multimedia Appendix 5).

##### Fixation Detection

To analyze fixations, ROIs will be defined for the 5 sound-source locations (L90, L45, C00, R45, and R90). For each ROI, we will extract fixation measures such as the total number and duration of fixations, horizontal amplitude of eye movement, total gaze path length, total explored area, last fixation duration and latency, and the name of the ROI containing the last fixation [78] (Table 5). The ROI containing the last fixation will serve as the primary eye-tracking outcome used to determine task performance.

**Table 5.** Measures used for the analysis of fixations. The regions of interest correspond to the silhouettes that are assumed to have emitted the sounds: L90, L45, C00, R45, and R90.

Description	Unit
Full image	
Total number of fixations	N/A^a^
Total fixation duration	Milliseconds
Horizontal amplitude of eye movement	Pixels
Total gaze path length	Pixels
Total explored area	Pixels
Last fixation	
Duration	Milliseconds
Latency	Milliseconds
Name of the ROI^b^ containing the fixation (if applicable)	N/A

a N/A: not applicable.

b ROI: region of interest.

Additionally, a data-driven analysis without predefined ROIs will be conducted using the iMap4 toolbox [79] to generate statistical fixation maps and validate the relevance of the predefined ROIs.

##### Pupil Diameter Calculation

Pupil data will be segmented into baseline-corrected epochs ranging from 300 milliseconds before sound onset to the end of image presentation and categorized according to sound spatial position, with the 300-milliseconds prestimulus interval used exclusively for baseline correction. Pupil responses will then be averaged sample-by-sample across identical sound positions. For each position, the following measures will be extracted over the 0‐ to 3.1-second poststimulus interval: mean pupil size (millimeters), maximum pupil size (millimeters), and latency to peak pupil dilatation (milliseconds).

##### Head Position

For each sound, head rotation along the azimuthal axis will be analyzed from the onset of the auditory stimulus until the end of the trial. For each epoch, the final head position will be computed in radians.

### Statistical Analysis

#### Overview

Statistical analyses will be conducted using RStudio. Statistical significance will be set at *P*<.05, with false discovery rate correction applied separately within each predefined family of analyses (eye tracking, head tracking, and pupillometry) [80]. Spearman correlation coefficients will be interpreted according to commonly used guidelines in the literature [81], distinguishing weak (*r*=0.10‐0.39), moderate (*r*=0.40‐0.69), and strong (*r*=0.70‐1.00) associations. A threshold of 75% will be used to indicate consensus, in line with commonly used approaches such as Delphi studies [67]. The management of missing eye tracking, head tracking, and pupillometry data is described in Multimedia Appendix 5. Participants with missing data for the strategy questionnaire, perceived difficulty ratings, age, or pure-tone audiometry will be excluded from the corresponding analyses, and the number of excluded participants will be reported.

#### Validity

For data analysis, the 5 sound positions will be reduced to 3 attentional areas (Sound Position variable): left (L90 + L45), center (C00), and right (R45 + R90). *Precision* will be assessed by comparing performance scores against the theoretical chance level associated with random allocation across spatial categories using 1-sided 1-sample Wilcoxon signed-rank tests [82]. Chance level was set according to the number of possible response locations: 40% for the left and right hemifields, corresponding to the probability of correctly identifying the left or right hemifield by chance (2 out of 5 possible response locations), and 20% for the central position, corresponding to the probability of correctly identifying the central position by chance (1 out of 5 possible response locations). Subsequently, an exploratory analysis will examine performance across the 5 original spatial positions (L90, L45, C00, R45, and R90) against the theoretical chance level of 20% (1 out of 5 possible response locations).

*Face and content validity* will be examined descriptively using frequencies, percentages, and 95% CIs for each item. Consensus will be evaluated based on the proportion of participants providing positive responses for each item. *Response process* evidence will be examined through four complementary sources: (1) the relationship between eye-tracking and head-tracking performance scores, as both response modes are intended to capture ASA, using Spearman rank correlation coefficient; (2) the strategies reported by participants to complete the assessment, analyzed using inductive thematic analysis, to determine whether they are consistent with ASA; (3) the impact of tracking order (eye tracking first vs head tracking first) on performance, assessed using the Mann-Whitney *U* test; and (4) the relationship between self-reported task difficulty and changes in pupil diameter, assessed using Spearman rank correlation coefficient, as pupillometry is assumed to reflect attentional load.

Because both sources of validity evidence rely on the same explanatory factors, *internal structure and fairness* will be examined simultaneously within 4 separate linear mixed-effects models: 2 binomial generalized linear mixed-effects models with a logit link function for eye-tracking and head-tracking performance, and 2 linear mixed-effects models for pupillary responses (mean and maximum pupil size). Sound position, background sound, tracking order, hearing score, and speech-in-noise score will be included as fixed effects where relevant, while participant will be included as a random effect to account for repeated measurements. Age group will be included only in the pupillometry models because of its known influence on pupil size and will be modeled as a 2-level categorical fixed effect (18‐40 years and 60‐80 years). See Table 6 for a detailed overview of the links between the study hypotheses and the planned validity analyses.

**Table 6.** Planned validity hypotheses, outcome measures, statistical analyses, and criteria supporting validity of study 2.

Validation domain	Statistical hypothesis	Statistical analysis	Result supporting validity
*Precision *(eye tracking)	Sound spatial area is correctly identified through gaze orientation	1-sided 1-sample Wilcoxon signed-rank tests	FDR^a^-corrected *P<.*05
*Precision* (head tracking)	Sound spatial areas are correctly identified through head orientation	1-sided 1-sample Wilcoxon signed-rank tests	FDR-corrected *P<.*05
*Face and content validity*	The assessment appropriately engages ASA^b^ using content adapted.	Frequency analysis	≥75% positive responses
*Response process* (eye vs head tracking)	Eye tracking and head tracking are expected to provide consistent measures of auditory spatial orienting	Spearman correlation	FDR-corrected *P*<.05; *Ρ*>.39
*Response process* (reported strategies)	Participants report strategies consistent with auditory spatial attention	Inductive thematic analysis and frequency analysis	≥75% strategies consistent with ASA
*Response process* (pupillometry)	Pupil dilation reflects perceived attentional demand	Spearman correlation	FDR-corrected *P*<.05; *Ρ*>.39
*Response process* (tracking order effect)	If both response modalities capture the same underlying auditory spatial attention processes, tracking order is not expected to influence performance	Mann-Whitney *U* test	Evidence of a meaningful tracking-order effect will be examined
*Internal structure*	Eye-tracking or head-tracking performance varies according to sound position and background sound	For head and eye movements separately: binomial generalized linear mixed-effects model with a logit link function; Performance∼Sound Position+Background+Tracking Order+Hearing Score+Speech-in-Noise Score+(1 | Participant)	Significant main effects of sound position and/or background sound (FDR-corrected *P*<.05), after accounting for tracking order, hearing score, and speech-in-noise score
*Fairness *(auditory measure)	Eye-tracking or head-tracking performance is not influenced by hearing abilities	For head and eye movements separately: binomial generalized linear mixed-effects model with a logit link function; Performance∼Sound Position+Background+Tracking Order+Hearing Score+Speech-in-Noise Score+(1 | Participant)	Potential associations between hearing score and performance, including interactions, will be examined
*Internal structure*	Attentional load is influenced by sound position and background sound	For mean and peak pupil size separately: mixed-effect model; Pupil Size∼Sound Position+Background+Tracking Order+Age Group+Hearing Score+Speech-in-Noise Score+(1 | Participant)	Significant main effects of sound position and background sound on pupil size (FDR-corrected *P*<.05), after accounting for tracking order, age group, hearing score, and speech-in-noise score
*Fairness *(age and auditory measures)	Pupillometry remains sensitive to attentional load regardless of age and hearing ability	For mean and peak pupil size separately: mixed-effect model; Pupil Size∼Sound Position+Background+Tracking Order+Age Group+Hearing Score+Speech-in-Noise Score+(1 | Participant)	Potential effects of age group, hearing score, and speech-in-noise score, including their interactions, will be examined.

a FDR: false discovery rate.

b ASA: auditory spatial attention.

#### User Experience

*Usability *will be analyzed using the same procedure as in study 1. *Satisfaction *will be analyzed using the same approach, with Likert-scale items analyzed as in study 1 and frequencies reported for closed-ended questions. In addition, study 2 will include an assessment of *sense of presence*. Participants are expected to perceive a great level of sense of presence. Median scores for each dimension and for the overall questionnaire will be compared against an agreement threshold of 4 (Agree), using 1-sample Wilcoxon signed-rank tests. A median score ≥4 will support no modification of the intervention.

## Results

This study is part of a broader doctoral research project that was funded in 2021 and officially launched in 2022. Recruitment for *study 1* (*expert clinicians*) began in November 2025 and was completed in January 2026. Data analysis is in progress, and the corresponding results are anticipated to be submitted for publication during the winter of 2026‐2027. Recruitment for *study 2* (*healthy participants*) began in March 2026. *As of July 2026*, 48 participants had been enrolled, and recruitment is expected to be completed in October 2026. Data analysis is expected to be completed, and the corresponding manuscript is anticipated to be submitted for publication in winter 2027‐2028. Any protocol modifications that may arise following step 1, particularly based on clinicians’ feedback, will be documented and reported in the final publications.

## Discussion

### Expected Findings

Although ASA impairments may affect many neurological populations, no validated assessment is currently available for routine clinical practice. We expect that this pilot study will provide (1) preliminary evidence supporting the validity of the developed ASA assessment and (2) information regarding user experience. These findings are intended to support the refinement of the prototype and inform the design of larger-scale studies for its validation. In this context, successful implementation is expected to be facilitated by the consideration of multiple factors from the earliest stages of development, including the characteristics of the technology, clinical settings, patients, clinicians, and health care policies [83,84].

### Comparison With Prior Work

Despite the relevance of physiological measures such as gaze orientation, head orientation, and pupil-size variation, as well as the potential of spatialized auditory environments delivered through headphones for assessing ASA, to our knowledge, these approaches have never been combined into a clinically implementable assessment. Nevertheless, recent advances in eye tracking and head tracking and auditory virtual reality technologies have increased their accessibility for clinical applications [85,86]. In contrast to existing ASA paradigms, which have primarily been developed for experimental research [1,6-9,11], the proposed assessment was specifically designed for clinical implementation. Furthermore, whereas eye tracking has already demonstrated value for assessing visual spatial attention and exploration [44], the present work extends its application to the assessment of ASA, potentially increasing the clinical usefulness of this technology across neuropsychological domains. Thus, the present work aligns with the growing movement toward digital neuropsychology while addressing the lack of clinically applicable tools for assessing ASA. Rather than simply digitizing an existing assessment, this approach uses technology both to create controlled auditory virtual environments and to capture objective physiological markers that are not readily accessible through conventional assessment methods. More broadly, the integration of digital assessments into routine neuropsychological practice is rapidly expanding because of their potential to improve assessment efficiency, provide objective behavioral measures, and support patient care [87,88].

### Limitations

However, we acknowledge the potential limitations of this study and encourage readers to interpret these analyses as preliminary. First, the sample size remains insufficient to support robust inferential analyses, and the single-center recruitment of healthy French-speaking participants limits the generalizability of the findings to broader clinical populations. Second, limitations related to psychometric evaluation should be noted. The exploratory analyses of age and hearing ability were designed to identify early indications that these variables may influence assessment performance and therefore warrant further investigation in subsequent validation studies. Detecting statistically significant differences at this preliminary stage would provide an early indication of potential fairness issues that should be examined in larger and more diverse samples. Conversely, the absence of statistically significant associations would not be interpreted as evidence of fairness or equivalence. Additionally, other factors such as hearing impairments and language should also be investigated to provide evidence related to fairness. Furthermore, as the assessment was administered during a single session, test-retest reliability could not be examined. However, this pilot study represents an essential and preliminary step in the validation process [20], and the limitations identified will be addressed in subsequent phases of the project. In particular, the digital nature of the assessment offers opportunities for personalization, including adaptations based on language and hearing impairment. Indeed, patients may fail the ASA assessment not because of an attentional impairment but because unilateral hearing impairments or interaural asymmetries alter their perception of auditory space. For this reason, this pilot study was restricted to participants with normal hearing in order to evaluate the assessment without the influence of peripheral auditory factors. However, whether hearing impairments significantly affect performance in the present auditory environment remains to be determined. Future studies will therefore be required to investigate these effects and, if necessary, develop hearing-informed calibration procedures, or adapt the sound-rendering algorithms to the patient’s hearing profile [89].

Third, the visual support may represent a limitation. Because the ASA assessment is intended for clinical settings, the selected tools need to remain as close as possible to those already commonly used in clinical practice. This led us to implement the visual environment required for eye-tracking measures on a standard computer screen. We acknowledge that this choice introduces a mismatch between the spatial coordinates of the auditory virtual environment and those represented by the silhouettes displayed on the computer screen. Nevertheless, two considerations supported this decision: (1) the objective of the assessment is to determine whether participants orient their attention toward broad spatial regions rather than to evaluate precise sound localization, so auditory stimuli were widely spaced while remaining within the anterior and lateral auditory space, and (2) alternative technologies such as HMDs and eye-tracking glasses were considered but were judged less suitable for clinical implementation. Future studies will be required to test this assumption directly within the ASA assessment and to determine whether the visual support influences performance in patients presenting with visual-spatial attention disorders.

### Futures Directions

If this pilot study provides conclusive evidence, the next phase will refine the prototype of the ASA assessment. Future work should involve a broader range of patient populations to further evaluate the validity, reliability, precision, and fairness of the assessment, as well as the need for accommodations or adaptations [20]. In addition, as the ultimate objective of the ASA assessment is to provide clinicians with a diagnostic tool for use with patients presenting neurological disorders, future studies will evaluate the characteristics required for medical device development and establish normative data based on a large sample of healthy participants. This step is particularly important, as many neuropsychological assessments, including attention measures, lack robust normative data, thereby limiting interpretive accuracy, and increasing the risk of false diagnoses of cognitive disorders [90]. At the present stage, none of the statistical analyses or effect sizes are intended to provide clinically interpretable thresholds or diagnostic indicators.

Over time, we hope that ASA assessment will become part of standard cognitive evaluation. Being able to assess ASA through a fast, simple, and systematic evaluation may contribute to a better understanding of its impact on daily functioning and of patients’ complaints across a range of clinical populations. For example, ASA impairments have been associated with learning difficulties in attention-deficit/hyperactivity disorder [91] and may contribute to difficulties in social learning among children with autism spectrum disorder [1]. Furthermore, some authors have suggested that ASA impairment may contribute to difficulties that are often attributed to other cognitive functions, such as working memory, in Alzheimer disease. Indeed, specific deficits in ASA have been reported in Alzheimer disease, independently of peripheral hearing loss or nonspatial auditory impairments [92], and may even emerge at early stages of the disease. It would also facilitate the initiation, replication, and extension of the limited number of studies currently available on ASA.

### Dissemination Plan

The findings of this study will be disseminated through peer-reviewed publications and presentations at national and international scientific conferences. Results will also be shared with clinicians, researchers, and other stakeholders involved in neuropsychological assessment and neurological rehabilitation.

## Supplementary material

10.2196/88231 Multimedia Appendix 1 Detailed auditory environment specifications.

10.2196/88231 Multimedia Appendix 2 Detailed procedural descriptions of each phase.

10.2196/88231 Multimedia Appendix 3 Posttest and postsession questionnaires for study 1.

10.2196/88231 Multimedia Appendix 4 Posttest and postsession questionnaires for study 2.

10.2196/88231 Multimedia Appendix 5 Detailed preprocessing and analysis pipeline for eye-tracking, pupillometry, and head-tracking data.

## Abbreviations

ASA: auditory spatial attention
HMD: head-mounted display
HRTF: head-related transfer function
I-CVI: item-level content validity index
ILD: interaural level difference
ITD: interaural time difference
ROI: region of interest
