# Supplementary material for: Eye Tracking–Based Evaluation of Auditory Spatial Attention in a Virtual Auditory Environment: Protocol for Development of a New Approach and Preliminary Validation
Source: JMIR Res Protoc. 2026 Sep 29;15:e88231. doi: 10.2196/88231 (PMC13623210; doi:10.2196/88231)
Supplement: Supplementary file 1 [file resprot-v15-e88231-s001.docx]

**Multimedia Appendix 1. Detailed auditory environment specifications**

Vocal sounds were recorded under controlled acoustic conditions and later converted into binaural format using the IRCAM SPAT convolution engine, which provides full head-related transfer functions (HRTFs). The auditory rendering was dynamically updated in real time according to each participant’s head orientation via the head-tracking system, coupled with MaxMSP software, which continuously varied the HRTFs (Figure S1).

The background sound was recorded in a Parisian coffee shop processed through the same convolution engine as the target sounds, using an ambisonic-to-binaural transformation to ensure realistic spatial rendering.

**Figure S1**: **Summary of recording, processing, and rendering of the auditory virtual environment.** Voices were recorded in mono and then dynamically converted into binaural according to control data (angle, distance, and head movement). The background was recorded in first-order ambisonics and then statically converted into binaural. The two binaural outputs were summed and then amplified through the headphones.

**Estimated end-to-end system latency**

End-to-end latency was not directly measurable under the experimental conditions. Instead, it was theoretically estimated by summing the reported or calculated latencies of the individual system components. The Supperware head tracker has a manufacturer-reported latency of less than 20 ms under the standard polling rate (50–52 Hz) ([https://supperware.co.uk/headtracker-overview](https://supperware.co.uk/headtracker-overview)). MIDI transmission of head-position data over USB was assumed to introduce negligible latency. Then, Max/MSP used IRCAM SPAT to spatialize the sound. Real-time binaural rendering was performed through the spat5.binaural~ MSP object within Max/MSP before direct audio output to the participant. According to Cycling ’74 documentation, latency in Max/MSP partly depends on signale vector size and sampling rate. In the present setup, the signal vector size used for real-time spatialization was 64 samples, with a sampling rate of 44,100 Hz. Following the formula proposed in the Max/MSP documentation (buffer size × 1000 / sampling rate), this corresponds to an estimated DSP processing latency of approximately 1.45 ms. Additionnaly, audio output to the headphones depended on the I/O vector size, which was also set to 64 samples. Using the same formula, the estimated audio-buffering latency was therefore approximately 1.45 ms. Finally, approximately 1 ms may be added by message reception and processing time (ie, the “event interval”) within Max/MSP when the Overdrive function is enabled ([https://docs.cycling74.com/userguide/scheduler/#scheduler-in-audio-interrupt](https://docs.cycling74.com/userguide/scheduler/#scheduler-in-audio-interrupt)).

Consequently, the theoretical motion-to-sound latency of the system was estimated at approximately 23.9 ms (20 + 1.45 + 1.45 + 1 ms), remaining close to or below the ~25 ms threshold previously reported for stable auditory spatial perception.

**Head-tracking blocks instructions**

Instructions used during the head-tracking blocks (Phase 2) were generated using AI-based speech synthesis through ElevenLabs. Voice generation used the French voice “Manuel” with the Eleven Multilingual v2 model. Parameters were set as follows: speech rate = 1.0, stability = 100%, similarity = 100%, style exaggeration = 50%, and MP3 output format at a sampling rate of 44.1 kHz.
