# Supplementary material for: Eye Tracking–Based Evaluation of Auditory Spatial Attention in a Virtual Auditory Environment: Protocol for Development of a New Approach and Preliminary Validation
Source: JMIR Res Protoc. 2026 Sep 29;15:e88231. doi: 10.2196/88231 (PMC13623210; doi:10.2196/88231)
Supplement: Supplementary file 2 [file resprot-v15-e88231-s002.docx]

**Multimedia Appendix 2. Detailed procedural descriptions of each phase**

This appendix presents the procedural details of each assessment phase, illustrates them with schematic representations, and describes the rationale behind the main methodological choices.

**Eye-Tracking Measures (Phases 1 and 2 of the ASA Assessment)**

For these measures, a visual display composed of five 2D silhouettes was used to indicate the perceived sound location. The centroid of each silhouette was computed from its binary mask using the Centroid property of the regionprops function in MATLAB. Each centroid was then associated with one of the five auditory target positions (-90°, -45°, 0°, +45°, and +90° azimuth). The resulting centroid coordinates and corresponding visual angles are reported in Table S1.

| Auditory Azimuth (°) | Silhouette | Centroid_X (px) | Centroid_Y (px) | HorizontalAngle_Centroid (rad) | VerticalAngle_Centroid (rad) |
| --- | --- | --- | --- | --- | --- |
| -90 | L90 | 146 | 988 | -0.351 | -0.173 |
| -45 | L45 | 452 | 504 | -0.225 | 0.043 |
| 0 | C00 | 960 | 242 | 0.000 | 0.160 |
| 45 | R45 | 1468 | 504 | 0.225 | 0.043 |
| 90 | R90 | 1774 | 989 | 0.351 | -0.173 |

Table S1. Centroid coordinates and corresponding visual angles of the five silhouettes. Centroid_X and Centroid_Y indicate the horizontal and vertical coordinates of the silhouette centroid in pixels, respectively. Coordinates were expressed relative to the upper-left corner of the display. HorizontalAngle_Centroid and VerticalAngle_Centroid indicate the corresponding horizontal and vertical visual angles of the centroid in radians, respectively. Visual angles were calculated relative to the center of the display. px = pixels; rad = radians.

**Habituation prior eye-tracking measurement (phase 1)**

The habituation phase was preferred over training for several reasons: (1) the response modes used (eye and head pointing) do not require specific learning since they are natural [1]; (2) we only intended participants to become familiar with the task, so habituation was sufficient, as training could affect the spontaneity of responses [2,3]; and (3) training phases are not suited to our objective, which is assessment rather than rehabilitation [4]. Habituation will be carried out in two steps, HA1 and HA2 (figure S2).

*HA1: Familiarization with the visual environment*

This first step comprises one trial with the visual environment only. Its goal is to allow participants to: (1) understand the mechanism linking the fixation cross to image display, (2) become familiar with the silhouettes and the participant’s position/disposition, and (3) provide a measure of free visual exploration of the image as a control measure of visual spatial attention.

First, a fixation cross appears, indicated by dynamic arrows displayed on the screen. The purpose of the dynamic arrows is to guide participants with visuospatial attention deficits to locate the fixation cross. Indeed, studies have shown that moving stimuli capture attention [5] as well as gaze [6]. These dynamic arrows will disappear after 200 ms of fixation on the cross. Then, the fixation cross itself will disappear after 2 s of fixation [7,8], giving way to the image. One of two images (woman or man silhouettes) will be randomly presented to the participants. They will be instructed to explore each silhouette within the entire image for 5 seconds. At the end, participants will be asked how many people they saw. Since eye orientation is closely linked to spatial localization and visual attention, this task provides an initial measure of their visual spatial attention abilities.

*HA2: Familiarization with the audiovisual environment*

This second step comprises at least one block of 5 trials (sounds) and up to three blocks of 5 trials (depending on the participant’s needs) in an audiovisual environment. The goals are: (1) to expose participants to the experimental phase conditions, and (2) to promote the association between each silhouette position and the corresponding vocal sound position.

For this purpose, 10 locutors (5 women and 5 men) will be randomly selected from the sound bank and excluded from the sounds presented to participants in Phases 2 and 4. Only the target vocal sound will be presented, without background sound. The visual environment will be the same as in HA1 (a fixation cross initially indicated by dynamic arrows, followed by the visual stimulus), except that it will now be accompanied by vocal sounds.

To allow participants to associate each sound position with a silhouette, one vocal stimulus (man or woman, with a first name) will be selected and repeated in order from the far right position (R90) to the far left position (L90), passing through R45, C00, and L45. At this stage, the experimenter initiates the trial by pressing the space bar, but participants must fixate on the cross to trigger the sound and image. Using the space bar allows the experimenter to adjust the pace and, if necessary, provide additional explanations before proceeding.

Participants will be instructed to locate the sound source by orienting their gaze toward the silhouettes and to maintain fixation on the identified silhouette until the image disappears.

**Figure S2: Representation of the two habituation steps (HA1 and HA2).** HA1: Step 1 (visual environment only). The screen successively displays (A) the fixation cross with dynamic arrows (disappearing after 200 ms of fixation), (B) the fixation cross alone (disappearing after 2 s of fixation), and (C) the silhouettes image (displayed for 3.1 s). HA2: Step 2 (audiovisual environment). Same sequence (A–C), but phase (C) is accompanied by voices sound (without background sound). The experimenter triggers the next trial with the space bar.

**Eye-tracking measurement (phase 2)**

Each participant completes four blocks (EE1, EE2, EE3, and EE4), two with background sound (coffee shop atmosphere) and two without background sound. The order of the blocks is randomized across participants. Each block consists of 10 trials, including two trials at each of the five sound positions (L90, L45, C00, R45, and R90). For each sound position, one trial uses a woman voice and the other a man voice. Within each block, the order of sound positions and voice gender is randomized.

At the beginning of each trial, participants are required to fixate the bottom-center cross. Dynamic arrows indicate the fixation cross and disappear once the participant has fixated it for 200 ms. Participants must maintain fixation on the cross for a total of 2 s. After this period, the image (silhouettes) is displayed for 3.1 s, during which the vocal stimulus (with or without background sound) is played. Participants are instructed to find the silhouette corresponding to the current talker and to maintain their gaze on that silhouette until the image disappears. They are also informed that the vocal sounds do not follow a fixed order, and their spatial positions vary randomly on each trial. When the image disappears, they return their gaze to the fixation cross in preparation for the next trial. Figure S3 shows this sequence.

**Figure S3: Representation of the experimental phase based on eye-tracking measurement (EE1 to EE4).** The screen successively displays (A) the fixation cross with dynamic arrows (disappearing after 200 ms of fixation), (B) the fixation cross alone (disappearing after 2 s of fixation), and (C) the vocal sounds together with the silhouettes image (displayed for 3.1 s) that participants are required to fixate.

**Head-Tracking Measures (Phases 3 to 5 of the ASA Assessment)**

**Habituation prior head-tracking measurement (phase 3)**

This phase comprises two trials in an audio-only environment, with participants keeping their eyes closed. The goal of the habituation phase prior to head-tracking measurement is to ensure that participants understand the instructions and the task.

To avoid any drift in head position, participants are instructed to return their head to a neutral position (0°) before the beginning of each trial. To achieve this, the eye tracker is used, and participants are instructed to fixate on the bottom-central fixation cross. Dynamic arrow cues are displayed until participants fixate the cross for 200 ms. The arrows then disappear, and fixation on the cross must be maintained for a total duration of 2 s. Next, a recorded voice (over headphones) instructs participants to close their eyes, orient their head toward the talker (a different voice) that will be presented, and maintain that orientation until another recorded voice prompts them to open their eyes and return their gaze to the fixation cross. The sequence then repeats. To verify that participants have their eyes closed at the beginning of each trial, two complementary verification methods are used: the eye tracker confirms that the eyes are indeed closed, and the head tracker ensures that the eye tracker’s signal loss is not simply due to head rotation rather than actual eye closure.

In this phase, participants complete two randomized trials: one with a woman voice and one with a man voice, presented from the L45 and R45 positions in randomized order, without background sound. The voices are randomly drawn from the same sound pool used in Phase 1. The L/R45 position was selected as it is more difficult to localize than L/R90, for which head-tracking can provide particular benefit. Figure S4 summarizes all the steps.

**Figure S4: Representation of the habituation phase prior to the head-tracking phase (HA3).** The task successively presents: (A) the fixation cross with dynamic arrows (disappearing after 200 ms of fixation), (B) the fixation cross alone (disappearing after 2 s of fixation), (C) the audio instruction to close the eyes and orient the head toward the sound, (D) the target vocal sound without background sound, toward which participants must orient within 3.1 s, and (E) the audio instruction to open the eyes, return gaze to the fixation cross, and return the head to a neutral position.

**Head-tracking measurement (phase 4)**

The procedure mirrors Phase 3 (head-tracking habituation)—that is, it takes place without any visual image, with head position and eyes closed controlled—but now includes all sound positions: L90, L45, C00, R45, and R90. Participants complete two randomized blocks (HE1 and HE2) of ten trials—one with background sound (coffee-shop atmosphere) and one without background sound. A pause is intended to check participant comfort and readiness. Key difference from the prior eye-tracking measurement phase (Phase 2): there are only two blocks (instead of four) of ten sounds, because (1) this is a secondary measure and (2) the task is more demanding. All positions are presented twice, once with a woman voice and once with a man voice—as in Phase 2, using voices drawn from the same sound pool. Figure S5 summarizes all the steps.

Similarly, participants were instructed to orient their head toward the talker and to keep their head oriented in that direction until the vocal instruction to open their eyes.

**Figure S5. Representation of the experimental head-tracking phases (HE1 and HE2).** The task successively presents: (A) the fixation cross with dynamic arrows (disappearing after 200 ms of fixation), (B) the fixation cross alone (disappearing after 2 s of fixation), (C) the audio instruction to close the eyes and orient the head toward the sound, (D) the target sound toward which participants must orient within 3.1 s, and (E) the audio instruction to open the eyes, return gaze to the fixation cross, and return the head to a neutral position.

**Head-movement baseline (phase 5)**

In block BH1, participants perform four trials consisting of alternating head rotations—two to the right and two to the left. Verbal instructions are delivered via headphones, prompting them to close their eyes and execute each movement as instructed. They are asked to rotate their head as far as possible to the right and then repeat the same movement to the left, twice in total. A 5-second interval is provided to allow participants to perform each movement. Figure S6 summarizes this phase.

**Figure S6: Representation of the head-movement baseline (BH1).**The task successively presents: (A) the audio instruction to close the eyes and orient the head as far as possible to the right, (B) the head movement itself with 5-second pause between next instruction, and (C) the audio instruction to orient the head as far as possible to the left. The sequence is repeated twice.

1. Populin LC. Human sound localization: measurements in untrained, head-unrestrained subjects using gaze as a pointer. Exp Brain Res. 2008;190:11‑30. https://doi.org/10.1007/s00221-008-1445-2

2. Carlini A, Bordeau C, Ambard M. Auditory localization: a comprehensive practical review. Front Psychol. 2024;15:1408073. https://doi.org/10.3389/fpsyg.2024.1408073

3. Neuhoff J. Ecological Psychoacoustics. BRILL; 2004.

4. Majdak P, Walder T, Laback B. Effect of long-term training on sound localization performance with spectrally warped and band-limited head-related transfer functions. J Acoust Soc Am. 2013;134:2148‑59. https://doi.org/10.1121/1.4816543

5. Smith KC, Abrams RA. Motion onset really does capture attention. Atten Percept Psychophys. 2018;80:1775‑84. https://doi.org/10.3758/s13414-018-1548-1

6. Itti L. CHAPTER 94 - Models of Bottom-up Attention and Saliency. In: Itti L, Rees G, Tsotsos JK, éditeurs. Neurobiology of Attention [Internet]. Burlington: Academic Press; 2005 [cité 20 oct 2025]. p. 576‑82. https://doi.org/10.1016/B978-012375731-9/50098-7

7. Andreu L, Sanz-Torrent M, Olmos JG, MacWhinney B. The formulation of argument structure in SLI: an eye-movement study. Clinical Linguistics & Phonetics. Taylor & Francis; 2013;27:111‑33. https://doi.org/10.3109/02699206.2012.751623

8. Wynn JS, Ryan JD, Buchsbaum BR. Eye movements support behavioral pattern completion. Proc Natl Acad Sci U S A. 2020;117:6246‑54. https://doi.org/10.1073/pnas.1917586117
