# Supplementary material for: Eye Tracking–Based Evaluation of Auditory Spatial Attention in a Virtual Auditory Environment: Protocol for Development of a New Approach and Preliminary Validation
Source: JMIR Res Protoc. 2026 Sep 29;15:e88231. doi: 10.2196/88231 (PMC13623210; doi:10.2196/88231)
Supplement: Supplementary file 3 [file resprot-v15-e88231-s003.docx]

**Multimedia Appendix 3. Post-test and post-session questionnaires for Study 1**

**Step 3 (post-test)**

**Face validity**

**Do you think the assessment was appropriate for measuring auditory spatial attention?**

☐Yes
☐No

Why ?

*Enter your response here:*

**User experience – satisfaction**

1. **I found the sound quality to be good.**
   *From 1 = strongly disagree to 5 = strongly agree (5-point Likert scale).*

☐ 1	☐ 2	☐ 3	☐ 4	☐ 5

2. **I found the voices easy to localize.**
   *From 1 = strongly disagree to 5 = strongly agree (5-point Likert scale).*

☐ 1	☐ 2	☐ 3	☐ 4	☐ 5

3. **I was able to perceive that the voices were presented from different spatial locations.**
   *From 1 = strongly disagree to 5 = strongly agree (5-point Likert scale).*

☐ 1	☐ 2	☐ 3	☐ 4	☐ 5

4. **During which block did you feel most comfortable localizing the sounds (eye tracking vs head tracking; with background noise vs without background noise)?** *(Open-ended)*

 ___________________________________________________________________________________

5. **Was one of the blocks more engaging or motivating to listen to than the others?** *(Open-ended)*

___________________________________________________________________________________

6. **What did you think about the background noise?** *(Open-ended)*

___________________________________________________________________________________

7. **The purpose of the visual environment was to support sound localization. What did you think about this visual environment?** *(Open-ended)*

___________________________________________________________________________________

8. **I found it easy to identify all the visual representations of the voices on the display.**
   *From 1 = strongly disagree to 5 = strongly agree (5-point Likert scale)*

☐ 1	☐ 2	☐ 3	☐ 4	☐ 5

**Step 4 (post-session)**

**Content validity (comprehensivness)**

Would you have liked the assessment or approach to include additional components? Please explain why and, if yes, specify which ones.

☐Yes
☐No

Why ?

*Enter your response here:*

**Content validity (relevance and clarity)**

For each item, please indicate how **relevant/clear** you found the following elements, where **1 = not at all relevant/clear** and **5 = extremely relevant/clear** (**one response only**). You may add a comment if you wish.

| **Items** | **1. Not at all relevant/clear** | **2** | **3** | **4** | **5. Extremely relevant/clea** | **Comment** |
| --- | --- | --- | --- | --- | --- | --- |
| 1. Sounds lateralized at 90° to the left and right | ☐ | ☐ | ☐ | ☐ | ☐ |  |
| 2. Sounds lateralized at 45° to the left and right | ☐ | ☐ | ☐ | ☐ | ☐ |  |
| 3. Sounds lateralized at 0° (center) | ☐ | ☐ | ☐ | ☐ | ☐ |  |
| 4. Visual silhouettes representing possible auditory positions | ☐ | ☐ | ☐ | ☐ | ☐ |  |
| 5. Silhouette representing the listener’s position | ☐ | ☐ | ☐ | ☐ | ☐ |  |
| 6. Presence of background sound (bar ambience) | ☐ | ☐ | ☐ | ☐ | ☐ |  |
| 7. Absence of background sound | ☐ | ☐ | ☐ | ☐ | ☐ |  |
| 8. Response modality: orienting the eyes toward the silhouette | ☐ | ☐ | ☐ | ☐ | ☐ |  |
| 9. Response modality: orienting the head toward the sound | ☐ | ☐ | ☐ | ☐ | ☐ |  |
| 10. Use of vocal targets (human voices) | ☐ | ☐ | ☐ | ☐ | ☐ |  |
| 11. Task duration | ☐ | ☐ | ☐ | ☐ | ☐ |  |
| 12. Number of targets | ☐ | ☐ | ☐ | ☐ | ☐ |  |
| 13. Image familiarization phase | ☐ | ☐ | ☐ | ☐ | ☐ |  |
| 14. Sound familiarization phase | ☐ | ☐ | ☐ | ☐ | ☐ |  |
| 15. Length of the instructions | ☐ | ☐ | ☐ | ☐ | ☐ |  |
| 16. Information provided in the instructions | ☐ | ☐ | ☐ | ☐ | ☐ |  |

**Would you like to elaborate on one or more of your responses?** *(Open-ended)*

*Enter your response here:*

**User experience – usability**

For each of the following statements, please indicate the extent to which you agree or disagree using a 5-point Likert scale ranging from 1 = strongly disagree to 5 = strongly agree (**one response only**).

| **Items du F-SUS** | **1. Pas du tout d’accord** | **2** | **3** | **4** | **5. Tout à fait d’accord** |
| --- | --- | --- | --- | --- | --- |
| 1. I think that I would like to use this system frequently. | ☐ | ☐ | ☐ | ☐ | ☐ |
| 2. I found the system unnecessarily complex. | ☐ | ☐ | ☐ | ☐ | ☐ |
| 3. I thought the system was easy to use. | ☐ | ☐ | ☐ | ☐ | ☐ |
| 4. I think that I would need the support of a technical person to be able to use this system. | ☐ | ☐ | ☐ | ☐ | ☐ |
| 5. I found the various functions in this system were well-integrated. | ☐ | ☐ | ☐ | ☐ | ☐ |
| 6. I thought there was too much inconsistency in this system. | ☐ | ☐ | ☐ | ☐ | ☐ |
| 7. I would imagine that most people would learn to use this system very quickly. | ☐ | ☐ | ☐ | ☐ | ☐ |
| 8. I found the system very cumbersome to use. | ☐ | ☐ | ☐ | ☐ | ☐ |
| 9. I felt very confident using the system. | ☐ | ☐ | ☐ | ☐ | ☐ |
| 10. I needed to learn a lot of things before I could get going with this system. | ☐ | ☐ | ☐ | ☐ | ☐ |

**Would you like to elaborate on one or more of your responses?** *(Open-ended)*

*Enter your response here:*

**User experience – intention to use**

For each of the following statements, please indicate the extent to which you agree or disagree, where 1 = strongly disagree and 5 = strongly agree (**one response only**). You may add a comment if you wish.

| **Items** | **1. Strongly disagree** | **2** | **3** | **4.** | **5. Strongly agree** | **Comment** |
| --- | --- | --- | --- | --- | --- | --- |
| 1. I would intend to use this approach in my clinical practice if it were available. | ☐ | ☐ | ☐ | ☐ | ☐ |  |
| 2. I believe this approach would provide substantial added value to my current assessments. | ☐ | ☐ | ☐ | ☐ | ☐ |  |
| 3. I believe this approach would be useful for better understanding my patients’ attentional difficulties. | ☐ | ☐ | ☐ | ☐ | ☐ |  |
| 4. If the equipment were available in my department or practice, I would use this approach on a regular basis. | ☐ | ☐ | ☐ | ☐ | ☐ |  |

**Would you like to elaborate on one or more of your responses?** *(Open-ended)*

*Enter your response here:*
