# Supplementary material for: Eye Tracking–Based Evaluation of Auditory Spatial Attention in a Virtual Auditory Environment: Protocol for Development of a New Approach and Preliminary Validation
Source: JMIR Res Protoc. 2026 Sep 29;15:e88231. doi: 10.2196/88231 (PMC13623210; doi:10.2196/88231)
Supplement: Supplementary file 4 [file resprot-v15-e88231-s004.docx]

**Multimedia Appendix 4. Post-test and post-session questionnaires for Study 2**

**Face validity**

**Did you feel that this test engaged your attention in space?**

☐Yes
☐No

**Why ?**

___________________________________________________________________________________

**Content validity**

1. **The silhouettes were well positioned and accurately represented the location of the voices.**

☐ 1	☐ 2	☐ 3	☐ 4	☐ 5

2. **The presence of the silhouettes helped me localize the sounds.**

☐ 1	☐ 2	☐ 3	☐ 4	☐ 5

3. **The listener’s position helped me.**

☐ 1	☐ 2	☐ 3	☐ 4	☐ 5

4. **The task duration was too long.**

☐ 1	☐ 2	☐ 3	☐ 4	☐ 5

5. **The training trials helped me.**

☐ 1	☐ 2	☐ 3	☐ 4	☐ 5

Circle the training trials that helped you:

6. **Would you have liked additional training on anything?**

☐Yes: Please explain: ________________________________________
☐No

7. **The instructions were clear.**

☐ 1	☐ 2	☐ 3	☐ 4	☐ 5

8. **Did the instructions provide enough detail to understand the task?**

☐Yes
☐No: Please explain: ________________________________________

9. **During the head-tracker assessment, were you bothered by the absence of visual support from the environment?**

☐Yes: Please explain: ________________________________________
☐No

**Response process validity**

1. **Among the voices, did any sentence or spoken first name cause difficulties for you?**

☐Yes: Which sentence(s) and/or first name(s)? _________________________
☐No

2. **Did you use any strategy to localize the sounds?**

☐Yes: What strategy(ies) did you use? _________________________
☐No

3. **The head-tracker phase with eyes closed was the easiest.**

☐ 1	☐ 2	☐ 3	☐ 4	☐ 5

4. **The eye-tracker phase with eyes closed was the easiest.**

☐ 1	☐ 2	☐ 3	☐ 4	☐ 5

**User experience – usability**

For each of the following statements, please indicate the extent to which you agree or disagree using a 5-point Likert scale ranging from 1 = strongly disagree to 5 = strongly agree (**one response only**).

| **Items du F-SUS** | **1. Pas du tout d’accord** | **2** | **3** | **4** | **5. Tout à fait d’accord** |
| --- | --- | --- | --- | --- | --- |
| 1. I think that I would like to use this system frequently. | ☐ | ☐ | ☐ | ☐ | ☐ |
| 2. I found the system unnecessarily complex. | ☐ | ☐ | ☐ | ☐ | ☐ |
| 3. I thought the system was easy to use. | ☐ | ☐ | ☐ | ☐ | ☐ |
| 4. I think that I would need the support of a technical person to be able to use this system. | ☐ | ☐ | ☐ | ☐ | ☐ |
| 5. I found the various functions in this system were well-integrated. | ☐ | ☐ | ☐ | ☐ | ☐ |
| 6. I thought there was too much inconsistency in this system. | ☐ | ☐ | ☐ | ☐ | ☐ |
| 7. I would imagine that most people would learn to use this system very quickly. | ☐ | ☐ | ☐ | ☐ | ☐ |
| 8. I found the system very cumbersome to use. | ☐ | ☐ | ☐ | ☐ | ☐ |
| 9. I felt very confident using the system. | ☐ | ☐ | ☐ | ☐ | ☐ |
| 10. I needed to learn a lot of things before I could get going with this system. | ☐ | ☐ | ☐ | ☐ | ☐ |

**Would you like to elaborate on one or more of your responses?** *(Open-ended)*

*Enter your response here:*

**User experience – satisfaction**

1. **I found the sound quality to be good.**
   *From 1 = strongly disagree to 5 = strongly agree (5-point Likert scale).*

☐ 1	☐ 2	☐ 3	☐ 4	☐ 5

2. **I found the voices easy to localize using eye orientation.**
   *From 1 = strongly disagree to 5 = strongly agree (5-point Likert scale).*

☐ 1	☐ 2	☐ 3	☐ 4	☐ 5

3. **I found the voices easy to localize using head orientation.**
   *From 1 = strongly disagree to 5 = strongly agree (5-point Likert scale).*

☐ 1	☐ 2	☐ 3	☐ 4	☐ 5

4. **I was able to perceive that the voices were presented from different spatial locations.**
   *From 1 = strongly disagree to 5 = strongly agree (5-point Likert scale).*

☐ 1	☐ 2	☐ 3	☐ 4	☐ 5

5. **Among the different sound positions shown on this sheet, did any of them cause difficulties for you?**

☐Yes
☐No

If yes, circle the position(s) that caused difficulties:

6. **With the help of the experimenter, answer the following question: Was one of the blocks more motivating to listen to? (Circle your answer)**

7. **The head-tracker phase with eyes closed was the easiest.**

☐ 1	☐ 2	☐ 3	☐ 4	☐ 5

8. **The eye-tracker phase with eyes closed was the easiest.**

☐ 1	☐ 2	☐ 3	☐ 4	☐ 5

9. **Was there a moment when you felt more comfortable localizing the sounds? If yes, circle your answer:**

10. **I enjoyed it when there was bar background sound.**

☐ 1	☐ 2	☐ 3	☐ 4	☐ 5

11. **I enjoyed it when there was no bar background sound.**

☐ 1	☐ 2	☐ 3	☐ 4	☐ 5

12. **I enjoyed the assessment using eye orientation.**

☐ 1	☐ 2	☐ 3	☐ 4	☐ 5

13. **I enjoyed the assessment using head orientation.**

☐ 1	☐ 2	☐ 3	☐ 4	☐ 5

14. **The bar ambience was motivating.**

☐ 1	☐ 2	☐ 3	☐ 4	☐ 5

15. **The voices were motivating.**

☐ 1	☐ 2	☐ 3	☐ 4	☐ 5

**Sense of presence**

For each of the following statements, please indicate the extent to which you agree or disagree, where 1 = strongly disagree and 5 = strongly agree (**one response only**).

| **Items** | **1. Strongly disagree** | **2** | **3** | **4.** | **5. Strongly agree** | **Comment** |
| --- | --- | --- | --- | --- | --- | --- |
| 1. I had the impression of being physically present in the environment. | ☐ | ☐ | ☐ | ☐ | ☐ |  |
| 2. I felt confident in this environment. | ☐ | ☐ | ☐ | ☐ | ☐ |  |
| 3. I had difficulty believing in this auditory environment. | ☐ | ☐ | ☐ | ☐ | ☐ |  |
| 4. I had the impression of experiencing what was happening in the environment. | ☐ | ☐ | ☐ | ☐ | ☐ |  |
| 5. I enjoyed the voices that were presented to me. | ☐ | ☐ | ☐ | ☐ | ☐ |  |
| 6. I enjoyed the bar background noise that was presented to me. | ☐ | ☐ | ☐ | ☐ | ☐ |  |
| 7. I felt that I was reacting to the presence of another person in the auditory environment. | ☐ | ☐ | ☐ | ☐ | ☐ |  |
| 8. While listening, I did not see time pass. | ☐ | ☐ | ☐ | ☐ | ☐ |  |
| 9. The environment resembled events that I have experienced. | ☐ | ☐ | ☐ | ☐ | ☐ |  |
| 10. I found the voices credible. | ☐ | ☐ | ☐ | ☐ | ☐ |  |
| 11. I found the bar ambience credible. | ☐ | ☐ | ☐ | ☐ | ☐ |  |
