# Supplementary material for: Eye Tracking–Based Evaluation of Auditory Spatial Attention in a Virtual Auditory Environment: Protocol for Development of a New Approach and Preliminary Validation
Source: JMIR Res Protoc. 2026 Sep 29;15:e88231. doi: 10.2196/88231 (PMC13623210; doi:10.2196/88231)
Supplement: Supplementary file 5 [file resprot-v15-e88231-s005.docx]

**Multimedia Appendix 5. Detailed preprocessing and analysis pipeline for eye-tracking, pupillometry, and head-tracking data**

All data preprocessing will be performed using MATLAB R2024a (MathWorks Inc., USA). Detailed description of the processing pipeline including 1) fixation detection, 2) pupil diameter calculation, and 3) head position. Figure S7 summarizes the eye-tracking data processing procedure.

**1) Fixation**

Fixations will be identified from the X and Y gaze coordinates recorded by the eye tracker using the MATLAB-based 2-Means Clustering toolbox (i2MC) developed by Hessels et al. [1]. This method will provide robust fixation detection even in the presence of signal noise or movement artifacts potentially associated with attentional fluctuations [2]. Default parameters will be used, except that the sampling frequency will be set to 120 Hz. To accommodate this sampling rate, we will use its parameters opt.downsamples = [2 3 5] and opt.chebyOrder = 7, as recommended by the authors.

This toolbox includes two main steps: missing data interpolation and fixation clustering. Missing data will be interpolated using the Steffen algorithm [3] when short gaps (≤100 ms) occurred, and fixations will then be detected using a 200-ms sliding window updated every 20 ms.

Fixation will then be labeled according to the sound presented at the time it occurs. Only fixations that take place between the onset of the sound and the end of the image presentation will be retained, corresponding to a total duration of 3.1 seconds, consistent with previous studies [4,5]. In line with the literature, fixations will be excluded if (1) their duration is shorter than 40 ms [1,6] or (2) they correspond to the first fixation following the fixation cross when it occurs near the fixation cross presented prior to image onset. Indeed, since no target silhouette will be displayed in this area, such fixations will be considered attentional remnants related to the cross and will therefore provide no relevant information about sound localization on the image [7]. The first fixation will then be discarded if it fell within ±10% of the screen center horizontally and at 30% of the screen height from the bottom. This region corresponds to the area used to compute the viewing duration of the fixation cross during the recording, prior to image presentation. In addition, other authors, in the context of visual spatial attention, also excluded the first 100 ms (thereby removing the first fixation), which is consistent with our approach [8].

**2) Pupil**

Pupil size data from the right and left eyes will be processed using the MATLAB toolbox *Pupil size* developed by Antonius Wiehler (<https://github.com/awiehler/pupil_dilation>, [9]). Pupil data processing will be first carried out on the entire recording [10].

Wiehler’s pipeline includes outlier rejection, interpolation of missing data, and bandpass filtering. To identify outlier rejection, Wiehler identifies as missing data any value that exceeds three median absolute deviation (MAD) from the median. Outliers and missing samples (e.g., blinks) will be interpolated using a piecewise cubic Hermite algorithm, and the data will be bandpass-filtered using the toolbox’s default parameters (high-pass cutoff = 1/128 Hz; low-pass cutoff = 1 Hz) [11–13].

Then, data from the right and left eyes will be averaged, and the recording will be segmented into epochs extending from 300 ms before sound onset to the end of the image presentation (3.1 s). This duration is consistent with previous studies [14,15]. Each epoch will correspond to one of four experimental conditions and will be baseline-corrected by subtracting the mean pupil value calculated during the [−300; 0] ms period [16]. Epochs will be labeled according to the spatial position of the sound (left, center, right), and pupil size variations will be averaged sample-by-sample across identical positions over the 0-3.1 s post-stimulus interval.

**3) Head position**

To reduce noise in measurements, a 4th-order low-pass Butterworth filter will be applied. The filter will initially be designed as a 2nd-order Butterworth filter and then applied using forward-backward filtering, resulting in an effective 4th-order filter. This technique will eliminate any phase distortion and will preserve the integrity of the signal’s phase response. The cutoff frequency will be set at 10 Hz, which will effectively preserve the signal dynamics while attenuating high-frequency noise [17]

Raw orientation signals (as also analyzed in their raw form by Stein et al. [18]) will be segmented into epochs corresponding to the experimental conditions. Each epoch will extend from sound onset to the end of the listening period (3.1 s), a time window consistent with previous studies [19].

Each epoch will be labeled according to the spatial position of the sound (left, center, right) and the head position at the end of the epoch will be used.

**Figure S7. Schematic representation of data processing pipeline for fixation detection and pupil size analysis**. *Note: the data shown were collected from a research team member during equipment verification and are not part of the experimental dataset*

1. Hessels RS, Niehorster DC, Kemner C, Hooge ITC. Noise-robust fixation detection in eye movement data: Identification by two-means clustering (I2MC). Behav Res. 2017;49:1802‑23. https://doi.org/10.3758/s13428-016-0822-1

2. Jostrup E, Claesdotter-Knutsson E, Tallberg P, Söderlund G, Gustafsson P, Nyström M. No Effects of Auditory and Visual White Noise on Oculomotor Control in Children with ADHD. J Atten Disord. SAGE Publications Inc; 2024;28:1668‑83. https://doi.org/10.1177/10870547241273249

3. Steffen M. A Simple Method for Monotonic Interpolation in One Dimension. Astronomy and Astrophysics. 1990;443‑50.

4. Thurlow WR, Mergener JR. Effect of stimulus duration on localization of direction noise stimuli. J Speech Hear Res. 1970;13:826‑38. https://doi.org/10.1044/jshr.1304.826

5. Gil-Carvajal JC, Cubick J, Santurette S, Dau T. Spatial Hearing with Incongruent Visual or Auditory Room Cues. Sci Rep. 2016;6:37342. https://doi.org/10.1038/srep37342

6. van Rooijen R, Junge C, Kemner C. No Own-Age Bias in Children’s Gaze-Cueing Effects. Front Psychol [Internet]. Frontiers; 2018 [cité 4 sept 2025];9. https://doi.org/10.3389/fpsyg.2018.02484

7. Doidy F, Desaunay P, Rebillard C, Clochon P, Lambrechts A, Wantzen P, et al. How scene encoding affects memory discrimination: Analysing eye movements data using data driven methods. Visual Cognition. Routledge; 2023;31:1‑17. https://doi.org/10.1080/13506285.2023.2188335

8. Kaufmann BC, Cazzoli D, Pflugshaupt T, Bohlhalter S, Vanbellingen T, Müri RM, et al. Eyetracking during free visual exploration detects neglect more reliably than paper-pencil tests. Cortex. 2020;129:223‑35. https://doi.org/10.1016/j.cortex.2020.04.021

9. Wiehler A, Branzoli F, Adanyeguh I, Mochel F, Pessiglione M. A neuro-metabolic account of why daylong cognitive work alters the control of economic decisions. Current Biology. 2022;32:3564-3575.e5. https://doi.org/10.1016/j.cub.2022.07.010

10. Kret ME, Sjak-Shie EE. Preprocessing pupil size data: Guidelines and code. Behav Res. 2019;51:1336‑42. https://doi.org/10.3758/s13428-018-1075-y

11. Chiossi JSC, Patou F, Ng EHN, Faulkner KF, Lyxell B. Phonological discrimination and contrast detection in pupillometry. Front Psychol. 2023;14:1232262. https://doi.org/10.3389/fpsyg.2023.1232262

12. Dan EL, Dînşoreanu M, Mureşan RC. Accuracy of Six Interpolation Methods Applied on Pupil Diameter Data. 2020 IEEE International Conference on Automation, Quality and Testing, Robotics (AQTR) [Internet]. 2020 [cité 4 sept 2025]. p. 1‑5. https://doi.org/10.1109/AQTR49680.2020.9129915

13. Medeiros J, Bernardes A, Couceiro R, Oliveira P, Madeira H, Teixeira C, et al. Optimal frequency bands for pupillography for maximal correlation with HRV. Sci Rep. Nature Publishing Group; 2025;15:3361. https://doi.org/10.1038/s41598-025-85663-2

14. Mathôt S, Vilotijević A. Methods in cognitive pupillometry: Design, preprocessing, and statistical analysis. Behav Res. 2023;55:3055‑77. https://doi.org/10.3758/s13428-022-01957-7

15. Nakakoga S, Shimizu K, Muramatsu J, Kitagawa T, Nakauchi S, Minami T. Pupillary response reflects attentional modulation to sound after emotional arousal. Sci Rep. Nature Publishing Group; 2021;11:17264. https://doi.org/10.1038/s41598-021-96643-7

16. Chevalier N, Meaney JA, Traut HJ, Munakata Y. Adaptiveness in proactive control engagement in children and adults. Developmental Cognitive Neuroscience. 2020;46:100870. https://doi.org/10.1016/j.dcn.2020.100870

17. Crenna F, Rossi GB, Berardengo M. Filtering Biomechanical Signals in Movement Analysis. Sensors [Internet]. Multidisciplinary Digital Publishing Institute; 2021 [cité 15 juin 2026];21. https://doi.org/10.3390/s21134580

18. Stein N, Watson T, Lappe M, Westendorf M, Durant S. Eye and head movements in visual search in the extended field of view. Sci Rep. Nature Publishing Group; 2024;14:8907. https://doi.org/10.1038/s41598-024-59657-5

19. Brimijoin WO, Boyd AW, Akeroyd MA. The Contribution of Head Movement to the Externalization and Internalization of Sounds. PLOS ONE. Public Library of Science; 2013;8:e83068. https://doi.org/10.1371/journal.pone.0083068
